# Comparison of Metatranscriptomic Samples Based on *k-*Tuple Frequencies

**DOI:** 10.1371/journal.pone.0084348

**Published:** 2014-01-02

**Authors:** Ying Wang, Lin Liu, Lina Chen, Ting Chen, Fengzhu Sun

**Affiliations:** 1 School of Information Science and Technology, Xiamen University, Fujian, China; 2 Molecular and Computational Biology, University of Southern California, Los Angeles, California, United States of America; Hospital for Sick Children, Canada

## Abstract

**Background:**

The comparison of samples, or beta diversity, is one of the essential problems in ecological studies. Next generation sequencing (NGS) technologies make it possible to obtain large amounts of metagenomic and metatranscriptomic short read sequences across many microbial communities. De novo assembly of the short reads can be especially challenging because the number of genomes and their sequences are generally unknown and the coverage of each genome can be very low, where the traditional alignment-based sequence comparison methods cannot be used. Alignment-free approaches based on *k*-tuple frequencies, on the other hand, have yielded promising results for the comparison of metagenomic samples. However, it is not known if these approaches can be used for the comparison of metatranscriptome datasets and which dissimilarity measures perform the best.

**Results:**

We applied several beta diversity measures based on *k*-tuple frequencies to real metatranscriptomic datasets from pyrosequencing 454 and Illumina sequencing platforms to evaluate their effectiveness for the clustering of metatranscriptomic samples, including three 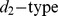 dissimilarity measures, one dissimilarity measure in CVTree, one relative entropy based measure *S2* and three classical 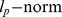 distances. Results showed that the measure 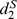 can achieve superior performance on clustering metatranscriptomic samples into different groups under different sequencing depths for both 454 and Illumina datasets, recovering environmental gradients affecting microbial samples, classifying coexisting metagenomic and metatranscriptomic datasets, and being robust to sequencing errors. We also investigated the effects of tuple size and order of the background Markov model. A software pipeline to implement all the steps of analysis is built and is available at http://code.google.com/p/d2-tools/.

**Conclusions:**

The *k*-tuple based sequence signature measures can effectively reveal major groups and gradient variation among metatranscriptomic samples from NGS reads. The 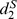 dissimilarity measure performs well in all application scenarios and its performance is robust with respect to tuple size and order of the Markov model.

## Introduction

The comparison of microbial communities is crucial for understanding how environment factors affect the composition and the function of the communities [1]. For the comparison of microbial communities, effective similarity/dissimilarity measures between the communities are urgently needed. Alignment-based sequence comparison methods such as the Smith-Waterman algorithm [2] and BLAST [3] have been the dominant approaches for the comparison of individual genomes when their genome sequences are available. However, microbial communities are usually complex and contain mixtures of hundreds to thousands of genomes with unknown genomic sequences. With the emergency of next generation sequencing (NGS) technologies, whole metagenome/metatranscriptome shotgun sequencing is becoming a new powerful approach to investigate complex microbial samples [4]–[13]. In metagenomic studies, the nucleotide DNA sequences are sampled and the composition of different organisms within the communities can be studied. On the other hand, in metatranscriptome studies, RNA sequences are sampled from the communities and the expression levels of various RNA molecules can be estimated. For both metagenomic and metatranscriptomic data, alignment-based approaches for the comparison of communities may not be applicable because the reads can be sampled from different parts of the genomes or RNAs of the various organisms. Alignment-based approaches are highly dependent on reliable reference sequences of known gene and/or pathway databases. However, most environmental microbial communities contain uncultured microorganisms, which affect the accuracy and completeness of alignment-based approaches. Therefore, alignment-free approaches provide attractive alternatives.

The *k*-tuple (*k*-word, *k*-gram) sequence signature of a community is defined as the frequency of *k*-tuples among the reads from the community. Previous studies have shown that *k*-tuple frequencies are similar across different regions of the same genome, but differ between genomes [14], which offers the theoretical basis to measure the dissimilarity between microbial communities with *k*-tuple method. The sequencing result of one microbial community is represented by a frequency vector of *k*-tuples, dependent only on the sequencing data, free from sequence alignments and reference genome information. Therefore, *k*-tuple based methods could potentially be very useful for the comparison of short reads data from NGS of microbial communities.

With the *k*-tuple sequence signature, each NGS data from a genome is represented by the *k*-tuple frequency vector whose elements are the number of occurrences of every *k*-tuple. With the *k*-tuple vectors, measurements of the dissimilarity between two genomic sequences were studied from different points of views, goals and applications. For two genomic sequences, the un-centered inner product of the two sequence signatures, 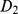, was first proposed to measure the distance of two *k*-tuple vectors [15], and then it was widely used in sequence database searches [16] and clustering of expressed sequence tags (ESTs) [17]. Various dissimilarity measures based on 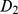 were extensively studied with different normalization, centralization and background models, including 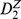 [18], 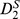 [19],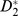 [20], *S2* [21], [22] and a measure in CVTree [23] (called *Hao* in this paper). However, the above measures were developed for evaluating the distance between two long sequences (such as genomic sequences). For better performance applying to NGS data, 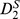 and 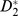 were further normalized to 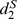 and 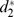 [24], respectively, with a range from 0 to 1, in order to reduce the effects of sequence length and different nucleotide probabilities.

The *k*-tuple sequence signatures have been applied to compare microbial communities based on metagenomic data [25]; study the evolutionary relationships among genomic sequences [26]; identify horizontal gene transfer among different genomes [27]; and bin genomic fragments from metagenomic samples [4], [28]. For the comparison of microbial communities based on NGS metagenomic datasets, Rohwer et al. [29] analyzed the ability of di-, tri- and tetra-nucleotide signatures to explain the variance between biomes and identified metagenomes with anomalous content. HabiSign [7] utilized patterns of tetra-nucleotide usage to cluster metagenomes at biome, phenotypic and species levels. Furthermore, Jiang et al. [25] applied 13 *k*-tuple based dissimilarity measures to compare metagenomic samples by clustering them into different groups as well as recovering environmental gradients affecting microbial samples, aiming to evaluate the performances of these measures on comparing metagenomic samples. They found that *k*-tuple sequence signatures can successfully reveal major group and gradient relationships among metagenomic samples from NGS reads without alignment to reference databases and the 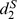 dissimilarity measure outperforms others in all application scenarios.

With the development of experimental and sequencing techniques, many metatranscriptomic datasets have been generated to explore the expressed genes and their abundances in microbial communities [5], [6], [9], [11], [13]. Metatranscriptomic data offers more elaborate details about what genes are expressed and their expression levels as well as the functions of the microbial communities and thus, it is crucial to compare metatranscriptomes among microbial environments. As far as we know, no alignment-free approaches have been used to compare metatranscriptomic data. Although our previous researches verified the effectiveness of alignment-free approaches on distinguishing the metagenomic datasets [25], it is built on the theoretical basis obtained by the previous studies that *k*-tuple frequencies are similar across different regions of the same genome, but differ between genomes [14]. When the target switches from DNA to RNA, the quantity and the structure of sequences are significantly changed. At the same time, the different characteristics of RNA from DNA, such as degradation, stability, easiness to be broken and alternative splicing, etc., bring different preferences and bias distributions to the sequencing. When the expression abundance information is imported and the sequences of intron and inter-genic regions are taken out, whether the alignment-free approaches are valid to distinguish the metatranscriptomic datasets is a critical question for their further applications to the metatranscriptomic datasets. Therefore, in this paper, we applied 16 *k*-tuple sequence signature measures to 99 metatranscriptomic and 16 metagenomic datasets from 13 communities/projects, among which 92 datasets from 12 communities were generated by the pyrosequencing 454 platform and 7 datasets from 1 community were generated by the Illumina Genome Analyzer IIx platform. The processing follows the same steps with our previous work [25]: *counting k-tuple vectors of each dataset, calculating signature measures between dataset pair and then clustering according to the dissimilarity matrix.* We conducted a series of computational experiments to study the effectiveness of the 16 *k*-tuple based sequence signature measures in clustering metatranscriptomic or mixture of metagenomic and metatranscriptomic datasets, identifying gradient relationships of microbial community samples, clustering ability when sequencing depth is low and the effect of sequencing errors on their performance. We also investigated the effects of various tuple sizes and the order of Markov model for the background genome sequences. We also developed a software pipeline to implement the processing procedures, which is more efficient in calculating, more comprehensive in function and more convenient to use compared to *d2Meta* for calculating the three d2-type measures in previous work [25] for analyzing metagenomic datasets.

## Materials and Methods

### Dissimilarity Measures based on *k*-tuple Sequence Signature

The sequence signature of a NGS data set counts the number of *k-*tuple occurrences within the reads. This representation makes the direct comparison of two sequence datasets, for example, two metatranscriptomic sequencing datasets, feasible. The comparison is free from alignment of the reads to reference sequences, which are often incomplete or unavailable. Therefore, in our paper, the sequence signature represented by *k-*tuple frequency is applied to compare metatranscriptomic datasets.

Without alignment to genome/transcriptome, the information of the reads’ strand direction cannot be obtained. Hence, we take both a read and its complement into consideration when counting *k-*tuple frequencies. For metagenomic or metatranscriptomic sequencing data, with four possible alphabet 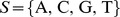, there are 4*^k^* possible tuples of length *k* in all reads. The numbers of occurrences in the whole sample for the *k-*tuples form the *k-*tuple frequency vector of the sample.

The *k-*tuple frequency vector represents the absolute number of occurrences of each *k-*tuple. For the distance between two datasets, proper normalization or centralization of the sequence signature is required. Therefore, there are various dissimilarity measures with different normalizations. In this paper, we studied various dissimilarity measures based on *k-*tuple frequency vectors, including three 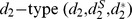 [24] dissimilarity measures, one dissimilarity measure in CVTree (called *Hao* in the paper) [23], one relative entropy based measure *S2* [22] and three classical 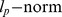 distances, Manhattan (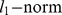), Euclidean (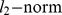) and Chebyshev (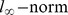). The detail of these dissimilarity measures can be found in the corresponding reference papers. For completeness, we briefly summarize the calculation for each measure as follows.

Let 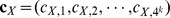 and 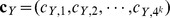 be the *k-*tuple frequency vectors from two NGS sequencing datasets 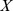 and 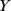, respectively. Let 
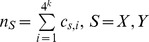
 be the sum of the counts of all *k-*tuples.

**The** 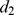 **dissimilarity measure** [24] is based on the 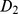 statistic [15] and is defined as:
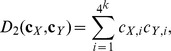


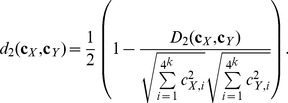
(1)


**The** 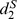 **and** 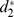 **dissimilarity measures** [24] are based on the 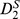 and 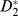 statistics [30] and they are defined as:
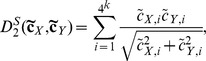


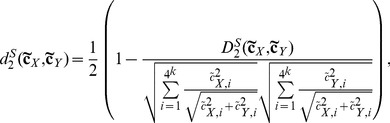
(2)and



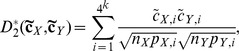


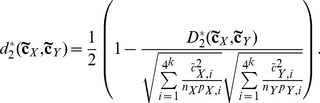
(3)


For formulas (2) and (3), 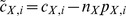 and 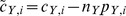 are centralizations, where 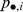 is the probability of the 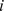-th *k-*tuple under a probability model (Markov model of order = 0–3) for the sequences. The ranges of 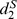 and 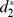 are between 0 and 1.

The Manhattan (***Ma***), Euclidean (***Eu***) and Chebyshev (***Ch***) dissimilarity measures are classical 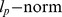 distances and are defined as:
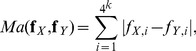
(4)

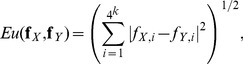
(5)

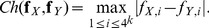
(6)

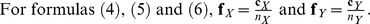



**The** ***Hao*** **dissimilarity measure** is from [23] by Bailin Hao’s group, so it is called *Hao* in our paper to simplify the notation. *Hao* is defined as follows:
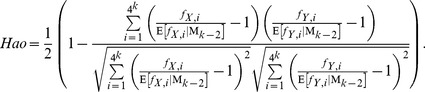
(7)


where 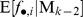 is the expectation of 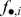 under the (*k*−2)-th order Markov chain.

**The** ***S2*** **dissimilarity measure** is a relative entropy based dissimilarity measure developed by Tianming Wang’s group [22]. *S2* is defined as:
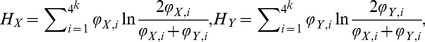
(8)

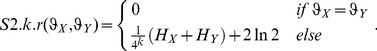
(9)


For formula (8), 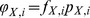 and 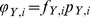, where, 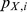 and 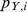 are the probability of the *i*-th *k*-tuple under an *r*-th order Markov chain for 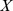 and 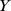, respectively.

#### The construction of Markov model [25]

Markov models of different orders were used to model the background sequences for the 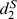, 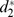, *S2* and *Hao* dissimilarity measures. Under Markov model 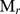 of order r, the probability of a *k*-tuple 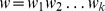, namely the expected frequency, can be computed as:
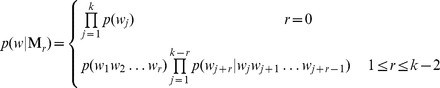
where 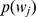 is the probability of 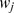 estimated by the ratio of the number of occurrences of 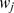 over all the number of nucleotides. The value of 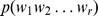 is estimated by the ratio of the number of occurrences of 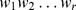 over all the number of *r*-tuple occurrences. The value of 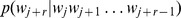 is estimated by the fraction of occurrences of 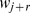 conditional on the previous occurrences of 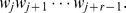


### Evaluation Metrics and Implementing Codes

**Symmetric difference** was originally defined to compare two sets. Robinson used symmetric difference as a criterion to evaluate the consistence between two trees [31]. For two trees with the same leaf nodes 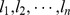, let A and B be the sets of nodes including the internal nodes of the two trees, respectively. Each node is denoted as a subset of their clustered leaves, that is, 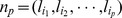. For tree nodes sets 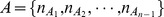 and 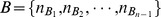, the symmetric difference is the number of nodes in 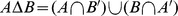, the union of nodes that are present in one tree but not in the other one, where 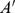 is the complement of set *A*. Actually, the symmetric difference is the number of nodes that are in one tree and not in the other one. Compared with Parsimony score [32], [33], symmetric difference takes the order of hierarchical clustering into consideration, which offers more meticulous comparison. The symmetric difference does not use branch length information, only the tree topologies. Hence, symmetric difference is applied to evaluate the consistence between the reference tree and the clustering tree in our study. Symmetric difference is calculated with Treedist from Phylip (http://evolution.genetics.washington.edu/phylip.html).

**UPGMA** (Unweighted Pair Group Method with Arithmetic Mean) [34] is used for hierarchical clustering based on dissimilarity matrix. Firstly, the dissimilarity between any two clusters A and B is calculated as the average of all dissimilarities between pairs of objects 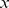 in A and 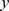 in B, written as: 
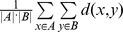
, where 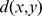 is the dissimilarity between 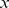 and 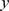. Then, at each step, the nearest two clusters are combined into a higher-level cluster. UPGMA is implemented with the function ‘upgma’ from the ‘phangorn’ toolbox of R.

**PCoA** (Principal Coordinates Analysis) [35] is also known as classical multidimensional scaling. If a dissimilarity matrix is denoted as 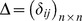, the objective of PCoA is to find 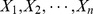, where 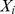 is a vector in an N-dimensional Euclid space, *N<n*, to optimize the function 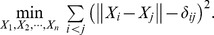 The results of PCoA are a set of eigenvalues and eigenvectors. The corresponding eigenvector of the largest eigenvalue is the first principal coordinate. The Goodness Of Fit (GOF) of PCoA reflects the accuracy that the coordinates approximate the distance matrix. The PCoA is implemented with the function ‘pcoa’ from the R ‘ape’ toolbox.

**Spearman’s Ranking Correlation Coefficient (SRCC)** assesses how well the relationship between two variables can be described using a monotonic function. If there are no repeated data values, a perfect SRCC of +1 or −1 occurs when each of the variables is a perfect monotone function of the other. In our study, SRCC is applied to evaluate the relationship between the gradient variable and the first principal coordinate of different measures. The SRCC is calculated by the function ‘cor’ from the R toolbox ‘stats’ of R.

#### The implementation software pipeline

We developed d2Tools with Python and R to count the *k*-tuple vectors, calculate probabilities of *k*-tuples under various orders of the Markov models and calculate the dissimilarity matrices under various dissimilarity measures 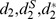, *Hao*, *S2*, *Ma*, *Eu* and *Ch*. The tool package can be downloaded from http://code.google.com/p/d2-tools/. Compared with *d2Meta*, the tool to implement the same processing steps for metagenomic comparison [25], *d2Tools* has the following improvements: 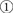 *d2Meta* only computes three d2-type (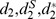) dissimilarity measures. For d2Tools, besides the three d2-type measures, *Hao, S2, Euclidean, Mahattan*, and *Chebyshev* distance measures are also included. 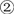 *d2Meta* only computes the probabilities of 0-order Markov model for d2-type measures, while *d2Tools* computes the probability and dissimilarity matrices on 0- to 3-order Markov model for these measures. 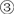 *d2Meta* accepts only the reads files as input and *d2Tools* accepts the reads files or the generated frequency and probability files by *d2Tools* as inputs. Therefore, users only need to keep the frequency of *k*-tuple files for future analysis. In addition *d2Tools* recognizes the input file format with the suffix name automatically.

We tested the ***d2Tools*** on the dataset including four samples (each sample is about 200 MB in size) in fasta format. There are a total of 2,830,286 reads [mean = 707,571±164,498(SD) per sample] and the read length is 164±102(SD) nt. It takes about 4 hours and 1.45 GB memory to finish the pipeline for 2–10 tuple sizes and 4 samples serially for all measures. The tuple sizes can be implemented simultaneously with simple shell command, which will speed up the running time of the pipeline. The running time of calculating the *k*-tuple frequency vector depends on the size of input datasets and the tuple size. The running memory mainly depends on the tuple size.

### Real Metatranscriptomic and Metagenomic Datasets

We downloaded 99 metatranscriptomic and 16 metagenomic datasets from 13 communities/projects in CAMERA (http://camera.calit2.net/) and NCBI SRA (http://www.ncbi.nlm.nih.gov/sra) databases. Ninety two metatranscriptomic and 16 metagenomic datasets from 12 communities were generated by the pyrosequencing 454 platform and 7 datasets from one community were generated by the Illumina Genome Analyzer IIx. There are a total of 16,453,499 reads with length 198±92 nt in the 92 metatranscriptomic datasets and 6,031,133 reads with length 443±212 nt in the 16 metagenomic datasets from the 454 sequencing platform. There are 11,447,063 reads with 76 nt in the 7 metatranscriptomic datasets from the Illumina platform. In the previous study of metagenomic comparison [25], 39 mammalian guts samples from 33 species (including omnivore, herbivore and carnivore samples) were analyzed the grouping ability of the sequence signature measures and 56 global ocean samples from 1 project were applied for grouping and gradient performance analysis. All the data in the previous study were metagenomic datasets generated with the pyrosequencing 454 platform. In our study, 92 metatranscriptomic ocean samples from 12 projects covering global ocean with different geographical locations, collection times, seasons and depths were applied to detect grouping and gradient performance for datasets from the pyrosequencing 454 platform. We also analyze the grouping characteristics when metagenomic and metatranscriptomic datasets co-exist using eight metagenomic and eight metatranscriptomic datasets. Furthermore, in order to explore the performance of sequence signature measures for different sequencing platforms, seven metatranscriptomic datasets of cecum and colon from the Illumina platform were analyzed.

**Figure 1:**
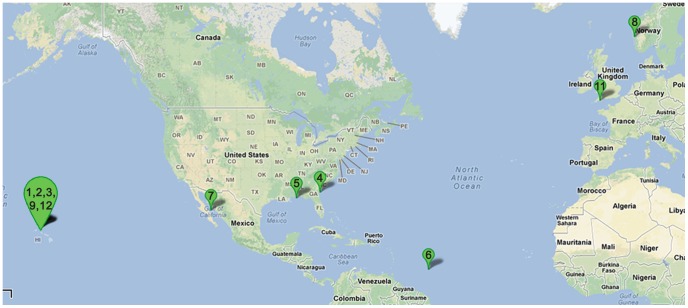
Geographical distribution of 11 communities in our study. There are 92 samples from 12 marine communities used in our study. ‘SWGE’, the Dataset 10 in Table 1, were collected from different locations with two research cruises in the Equatorial North Atlantic ocean and South Pacific Subtropical gyre. The locations of the other 11 communities are marked on the above map (using the DatasetID from Table 1), where we can find that Datasets 1,2,3,9,12 are collected from nearby locations.

The brief summaries of the datasets of the 13 projects are shown in Table 1 and the locations of 11 marine communities are marked in Figure 1.

**Table 1:** Description of datasets from 13 communities/projects used in this study.

Dataset ID & Name	Sequencing Platform	Database	Accession number in Database	Type and Number of Samples
1. Hawaii_Aug_DOM [10]	454	CAMERA	CAM_P_0000715	7 MT (4 control &3 Amended)
2. Hawaii_Aug_DSW	454	CAMERA	CAM_P_0000766	10 MT (5 control & 5 Amended)
3. Hawaii_Nov_Day Night [12]	454	CAMERA	CAM_PROJ_DayNight	2 MT
4. Georgia [11]	454	CAMERA	CAM_PROJ_CAM_P0000101	6 MT (2 control & 2 PUT & 2 SPD)
5. Mexico	454	CAMERA	CAM_PROJ_DICE	4 MT (2 control & 2 Amended)
6. Eastern Equa Atlan_Amazon	454	CAMERA	CAM_PROJ_AmazonRiverPlume	19 MT
7. California_Deep sea [5], [6]	454	CAMERA	CAM_P_0000545	4 MT
8. Norway	454	CAMERA	CAM_PROJ_PML	4 MT (2 control & 2 Amended)
9. Hawaii_depth	454	CAMERA	CAM_PROJ_HOT	4 MT
10. SWGE	454	CAMERA	CAM_PROJ_GeneExpression	16 MT
11. WesternEnglish	454	CAMERA	CAM_PROJ_WesternChannelOMM	8 MT & 8 MG
12. NPSG [13]	454	NCBI SRA	SRA007802.3/SRA007804.3/	4 MG×2 replicates
			SRA007806.3/SRA000263/	&
			SRA007801.5/SRA000262/	4 MT×2 replicates
			SRA007803.3/SRA007805.4	
13. Mouse_Intestinal [36]	Illumina	NCBI SRA	SRA051354	7 MT

MT: Metatranscriptomic data; MG: Metagenomic data; PUT: Putrescine; SPD: Spermidine; DOM: Dissolved Organic Matter; DSW: Deep SeaWater. The 12 datasets are collected from CAMERA and NCBI SRA datasets. Some of them have corresponding datasets, others are submitted to datasets. The first column indicates the symbol name in this paper.

#### Datasets in Experiment 1

All the 92 metatranscriptomic datasets from the pyrosequencing 454 platform in Table 1 were analyzed with the various dissimilarity measures. The objective is to evaluate their performance of grouping different samples/communities. First, 19 metatranscriptomic datasets from 4 different geographical marine locations (Dataset 2,4,7,11 in Table 1) were studied. There are 6,633,683 total reads [mean = 349,141±258,620(SD) per sample]. The read length is 180±94(SD) nt. These four communities are located on subtropical north Pacific (Hawaii), north Atlantic (West English Channel), foot of north Atlantic (Sapelo Island) and East Pacific ocean (Gulf of California). They are geographically separated distinctively and sampled in May or August, from the surface or deep sea hydrothermal plumes. Therefore, the reference cluster of their grouping is clear. With *k-*tuple vectors, different measures are applied to calculate the dissimilarity between sample-pairs. Based on the dissimilarity matrix, the different samples are hierarchically clustered using UPGMA [34]. The symmetric difference scores between the derived clusters and the reference cluster are calculated. Second, with obtained optimal *k*, Markov model and dissimilarity measures yielding the lowest symmetric difference, the entire 92 metatranscriptomic datasets from 12 communities/projects are clustered to see the performance of corresponding measures.

To evaluate the effect of sequencing depth on the dissimilarity between metatranscriptomic sample-pairs, the 19 metatranscriptomic datasets of 4 communities were sampled with different rates. For each sample, 10%, 1% and 0.1% of reads are sampled randomly for 100 times, and the averaged symmetric differences between the clustering results of the sampled reads and the reference cluster are calculated to assess the effect of sequencing depth under different dissimilarity measures.

#### Datasets in Experiment 2

The objective of Experiment 2 is to evaluate the performance of these measures in recovering the gradient relationships among the samples, that is, the change of gene expression along a gradient such as ocean depth, temperature, or pH level. Eight metatranscriptomic data sets from 25 m, 75 m, 125 m and 500 m depth (Dataset 12 in Table 1, two samples for each depth) of North Pacific Subtropical Gyre (NPSG) in ALOHA stations are used. Except for the depth, other collecting conditions are the same. Therefore, they are depth-gradient metatranscriptomic datasets. There are a total of 451,482 reads [mean = 56,435±7,701(SD) per sample]. The read length is 122±17(SD) nt. Based on the dissimilarity matrices under different settings and measures, PCoA is carried out to find the principal coordinate. Then the SRCC is calculated between the first principal coordinate and the pre-determined depth-gradient axis. A higher SRCC indicates better performance in identifying the gradient among the metatranscriptomic samples.

For these datasets, we also randomly sample them with 10%, 1% and 0.1% rates for 100 times. The PCoA and SRCC are also calculated to evaluate the measures’ ability to identify the gradient datasets under different sequencing depth.

#### Datasets in Experiment 3

The objective of Experiment 3 is to evaluate the ability of the dissimilarity measures to separate metagenomic from metatranscriptomic datasets. Eight metatranscriptomic and eight corresponding metagenomic datasets of two communities, respectively (Datasets 11 and 12 in Table 1), are used for this experiment. For dataset 11, there are 1,116,440 [mean = 139,555±28,192(SD) per sample] reads from the 8 metatranscriptomic data and the read length is 295±176(SD) nt; and there are 4,504,724 reads from the 8 metagenomic data [mean = 563,090±126,132(SD) per sample] and the read length is 555±108(SD) nt. For dataset 12, there are 451,482 reads [mean = 56,435±7,701(SD) per sample] from the 8 metatranscriptomic data and the read length is 122±17(SD) nt; and there are 1,526,409 reads [mean = 190,801±12,622(SD) per sample] from the 8 metagenomic data, and the read length is 115±12(SD) nt. The separation characteristics of these measures are explored based on the hierarchical clustering results.

To evaluate the effect of low sequencing depth, we sample the 16 metagenomic and metatranscriptomic datasets with 10%, 1% and 0.1% rates for 100 times to see the corresponding clustering results.

#### Datasets in Experiment 4

Currently, most metatranscriptomic datasets are produced by the pyrosequencing 454 platform. With the decrease of Illumina sequencing cost, some researchers began to sequence metatranscriptomic datasets with the Illumina platform. The objective of Experiment 4 is to evaluate the ability of the dissimilarity measures to separate metatranscriptomic communities from Illumina datasets. The datasets (Datasets 13 in Table 1) in this experiment are the metatranscriptomic samples from mouse cecum and colon [36], generated by the Genome Analyzer IIx (GaIIx) platform. There are 12 datasets from 7 samples with two RNA extraction protocols, Qiagen-based protocol and Invitrogen protocol. In order to obtain clear clustering ground truth, we extracted 7 samples produced with the Qiagen protocol. They are NOD501CecQN, NOD501ColQN, NOD502CecQN, NOD502ColQN, NOD503CecQN, NOD504CecQN and NOD504ColQN, where the digital numbers, such as ‘501’ are the mouse ID, ‘Cec’ means cecum and ‘Col’ means colon, QN means Qiagen-based protocol. In the seven metatranscriptomic datasets, there are 11,447,063 [mean = 1,635,294±333,882(SD) per sample] and the read length is 76 nt.

To evaluate the effect of low sequencing depth, we sample the seven metagenomic datasets with 1%, 0.1% and 0.01% rates for 100 times to see the corresponding clustering results.

#### Datasets in Experiment 5

The objective of this experiment is to study the effect of sequencing errors on the performance of these measures. The same 19 metatranscriptomic datasets from 4 different geographical marine locations (Datasets 2,4,7, and 11 in Table 1) in Experiment 1 were used in this experiment. The 19 datasets were considered as complete correct sequencing data. According to the characteristics of pyrosequencing 454, 1% indel and 0.1% substitution errors are imported to the datasets with FlowSim [37], a simulation software, and output simulated reads with pre-setting error rate and error models. Compared with the clustering results of the original datasets, the effects of sequencing errors on the performance of these dissimilarity measures are studied.

## Results

The real data are used to analyze the effectiveness of *k*-tuple based sequence signature measures for the comparison of microbial community samples. We studied the performance of 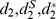, *Hao*, *S2*, *Ma*, *Eu* and *Ch* dissimilarity measures.

### Experiment 1: The Performance of Different Dissimilarity Measures using Sequence Signatures for Clustering Global Ocean Metatranscriptomic Datasets

Ninety-Two metatranscriptomic data collected from global ocean by twelve different projects were sequenced with the pyrosequencing 454 platform and were downloaded from CAMERA and NCBI SRA. The data are from different geographic locations including Hawaiian Ocean, Mexican Gulf, California Gulf, Norwegian Fjord, North Atlantic ocean, South Pacific ocean, Western English Channel and Eastern Equatorial Atlantic Ocean mixed with Amazon river plume. The descriptions and datasets ID can be found in Table 1.

**Figure 2:**
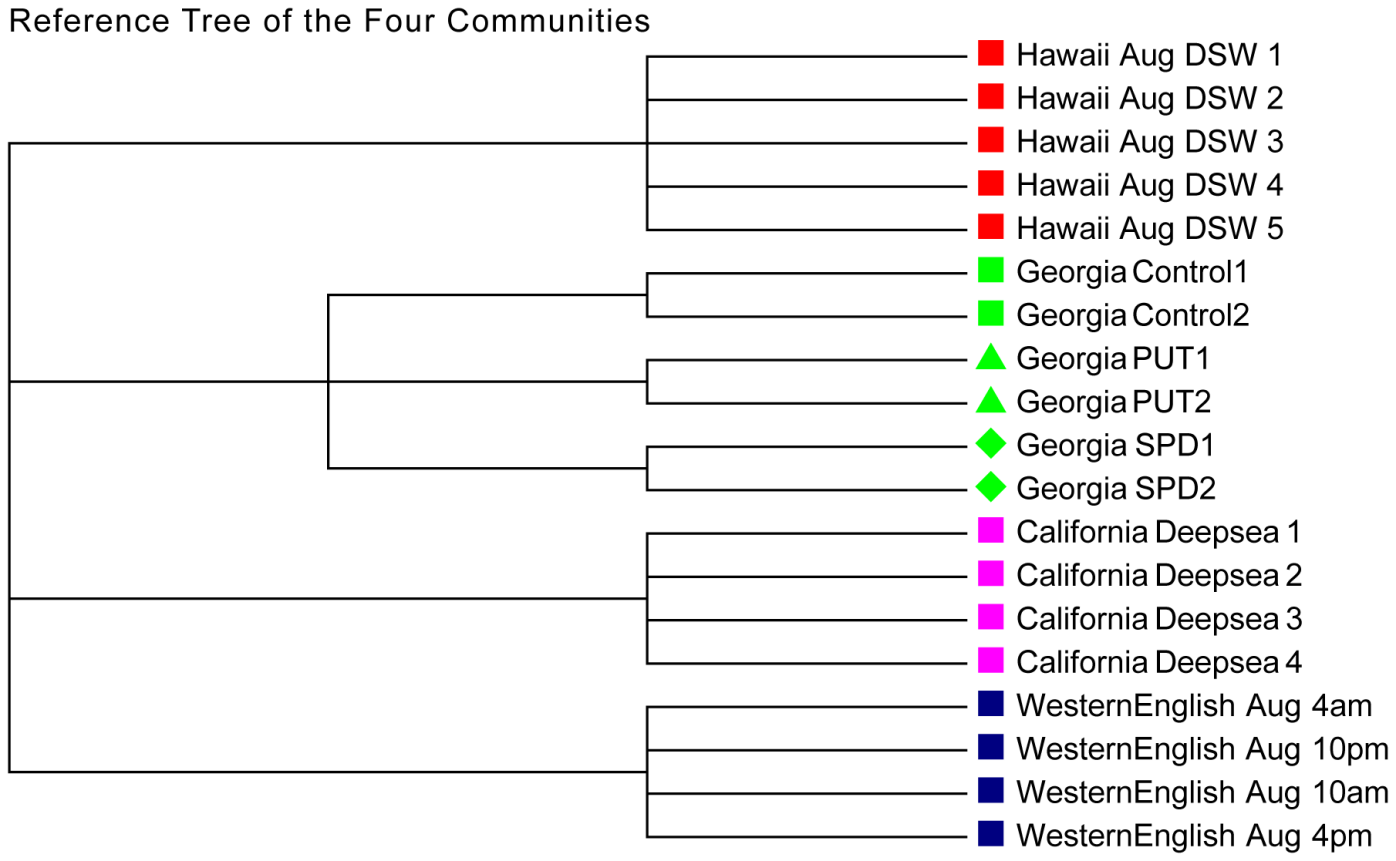
The reference tree of the four communities in Experiment 1 (without branch length information). The four communities are located at four distinct geographical locations with clear clustering characteristics. For the Georgia data, there are two control and two PUT (Putrescine) experimental and two SPD (Spermidine) experimental datasets.

First, 19 metatranscriptomic samples of 4 communities that have clear grouping relationships were extracted. These four communities are geologically separated distinctively, located on subtropical north Pacific (Hawaii), north Atlantic (West English Channel), foot of north Atlantic (Sapelo Island) and East Pacific ocean (Gulf of California). So the 19 samples can be clustered into 4 groups distinctively. Furthermore, Dataset 4 (Georgia_May) contains 2 control samples, 2 PUT-amended samples and 2 SPD-amended samples. Therefore, within the same community, the samples from the same condition should merge first. The reference clustering tree for the 19 samples from the 4 communities is shown in Figure 2, without distance information included. Based on the *k-*tuple frequency vectors and the 16 dissimilarity measures, the dissimilarity between each sample-pair is obtained. With the dissimilarity matrix, hierarchical clustering is implemented with UPGMA. The symmetric differences between the clustering results and reference cluster under various measures, tuple size and order of Markov model are calculated as shown in Table 2.

**Table 2:** Symmetric differences between clustering and reference tree for the four communities in Experiment 1.

*k*	2	3	4	5	6	7	8	9	10
*d*_2_	16	16	16	16	14	14	***12***	***12***	***12***
*d*_2_^*s*^|M_0_	18	14	16	16	***12***	***12***	***12***	14	14
*d*_2_^*s*^|M_1_	14	16	16	16	***12***	***12***	***12***	***12***	14
*d*_2_^*s*^|M_2_	NA	14	16	16	***12***	***12***	***12***	***12***	14
*d*_2_^*s*^|M_3_	NA	NA	14	16	***12***	***12***	***12***	***12***	14
*d*_2_*|M_0_	18	14	16	16	***12***	***12***	***12***	***12***	***12***
*d*_2_*|M_1_	14	14	16	16	***12***	***12***	***12***	***12***	***12***
*d*_2_*|M_2_	NA	14	16	16	***12***	***12***	***12***	***12***	***12***
*d*_2_*|M_3_	NA	NA	14	***12***	***12***	***12***	***12***	***12***	***12***
*S2*|M_0_	22	22	18	18	18	18	18	18	16
*S2*|M_1_	22	20	18	18	18	18	18	18	16
*S2*|M_2_	NA	18	18	18	18	18	18	18	16
*Hao*	NA	14	16	16	14	14	***12***	***12***	14
*Ma*	16	16	16	18	18	***12***	***12***	14	14
*Eu*	16	16	18	18	16	16	16	14	14
*Ch*	16	22	18	22	20	20	18	20	22

For all scores, p-value<0.001. Each column is the symmetric differences when tuple size is from 2 to 10. Each row gives the symmetric differences for a given dissimilarity measure and *d*_2_^*s*^|M_0_ means *d*_2_^*s*^ measure under 0-th order Markov model. Others symbols have similar meaning.

**Figure 3:**
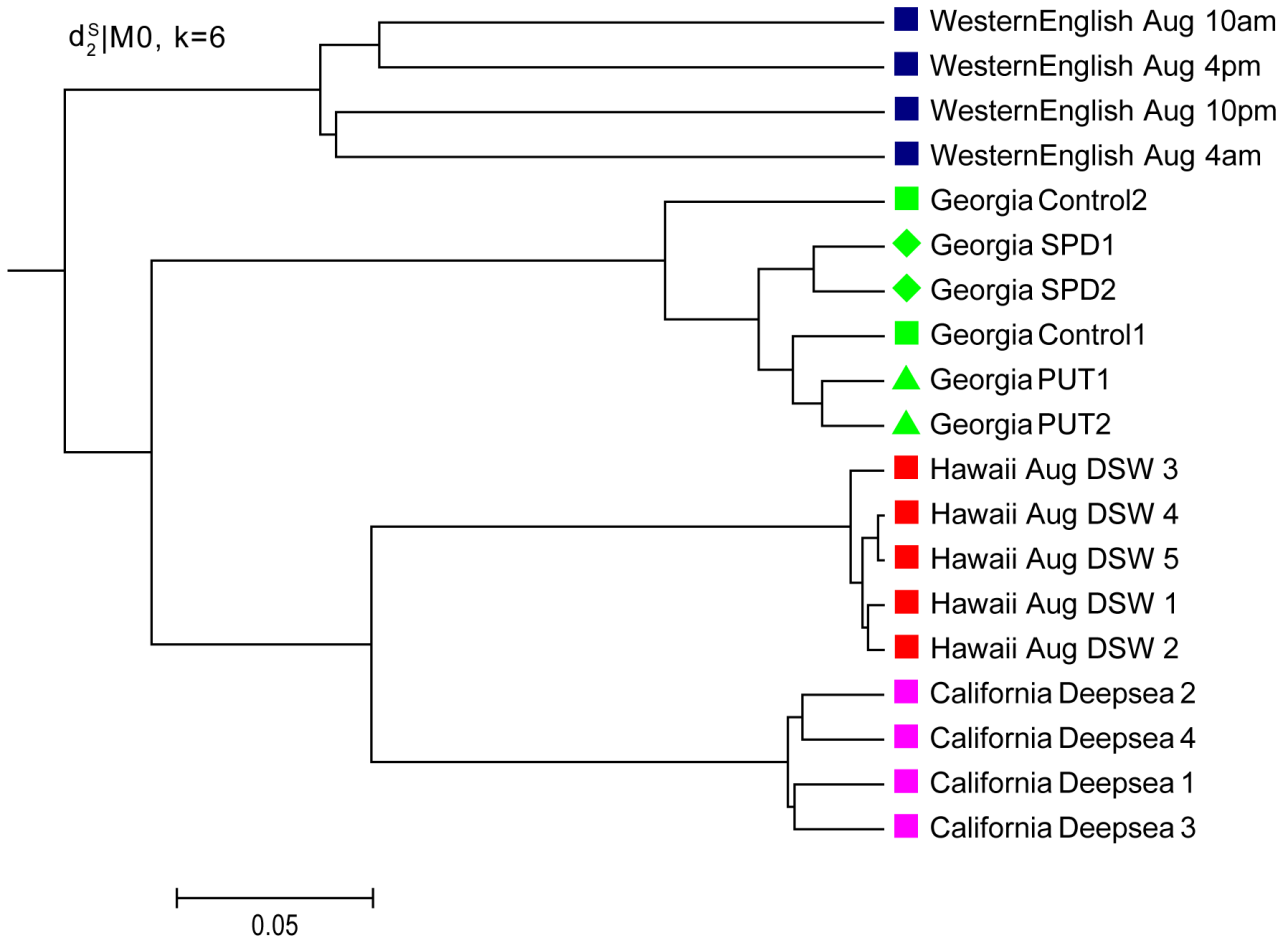
Clustering results of the four distinctive communities in Experiment 1 based on *d*_2_^*s*^|M_0_ and k = 6. *d*_2_^*s*^|M_0_ indicates using dissimilarity measure based on 0-th order Markov chain model. All the basic clusters for the four communities are correct. For the sub-classes in the Georgia communities, except for the two control samples, the SPD and PUT sub-classes are clustered correctly.

In Table 2, the optimal symmetric difference score between the reference tree and clustering results is 12. Almost all the dissimilarity measures except *S2*, *Eu* and *Ch* can achieve this optimum score with appropriate choices of tuple size *k*. However, the range of the values *k* that yields the optimal results is different. For 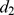 the optimal score is obtained for k = 8, 9, 10. For 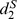, the optimal results are obtained when *k* is between 6–9 with the order of the Markov chain not significantly affecting the results. For 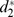, the optimal results are obtained for *k* between 6–10 for all orders of the Markov chain. Thus, the results are robust with respect to the length of *k*-tuples and the order of Markov chain. We observed that big ranges of settings under 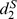 and 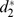 measures and certain settings under *Hao* yield the optimal value. For 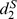 and 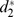, the optimal clustering trees are all obtained from 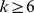 for 0–3rd orders of Markov model. The order of background Markov Model has less effect on the clustering results. One of the optimal clustering trees with k = 6 and 0-th order Markov model under 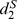 is shown in Figure 3. Except for the sub-classes of two control samples from the Georgia_May data, all the basic groups of four communities are clustered well. It is also observed that the communities with close geographical locations are clustered first. Because the latitudes of Hawaii and California are very close, they are clustered first, and then the second closest Georgia May channel is merged. The farthest West English Channel joins at last. This clustering order reflects that the communities with similar geographical conditions are more similar with respect to their gene expression levels, which also fit the biological intuition.

**Figure 4:**
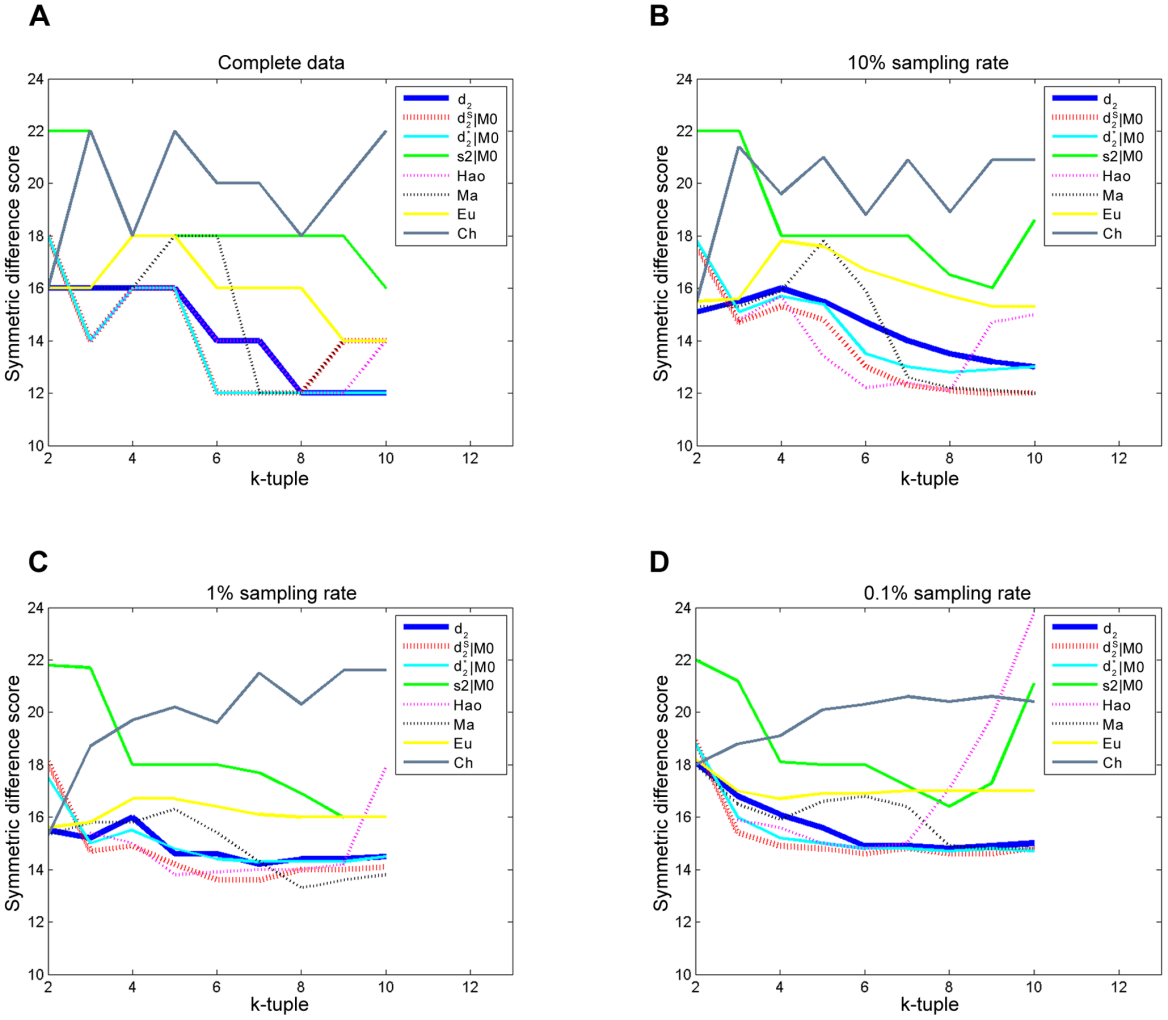
Average symmetric difference scores for the four distinct communities under different sampling rates in Experiment 1. (A) is the symmetric difference scores for complete data as a function of tuple size *k* for different dissimilarity measures. (B) (C) (D) are the average symmetric difference scores as a function of tuple size k for different dissimilarity measures after 100 random samplings for 10%, 1% and 0.1% sampling rates, respectively. The lower the score is, the closer the clustering results and reference tree are. It is clear that *d*_2_^*s*^ shows the best performance under most of the conditions.

**Figure 5:**
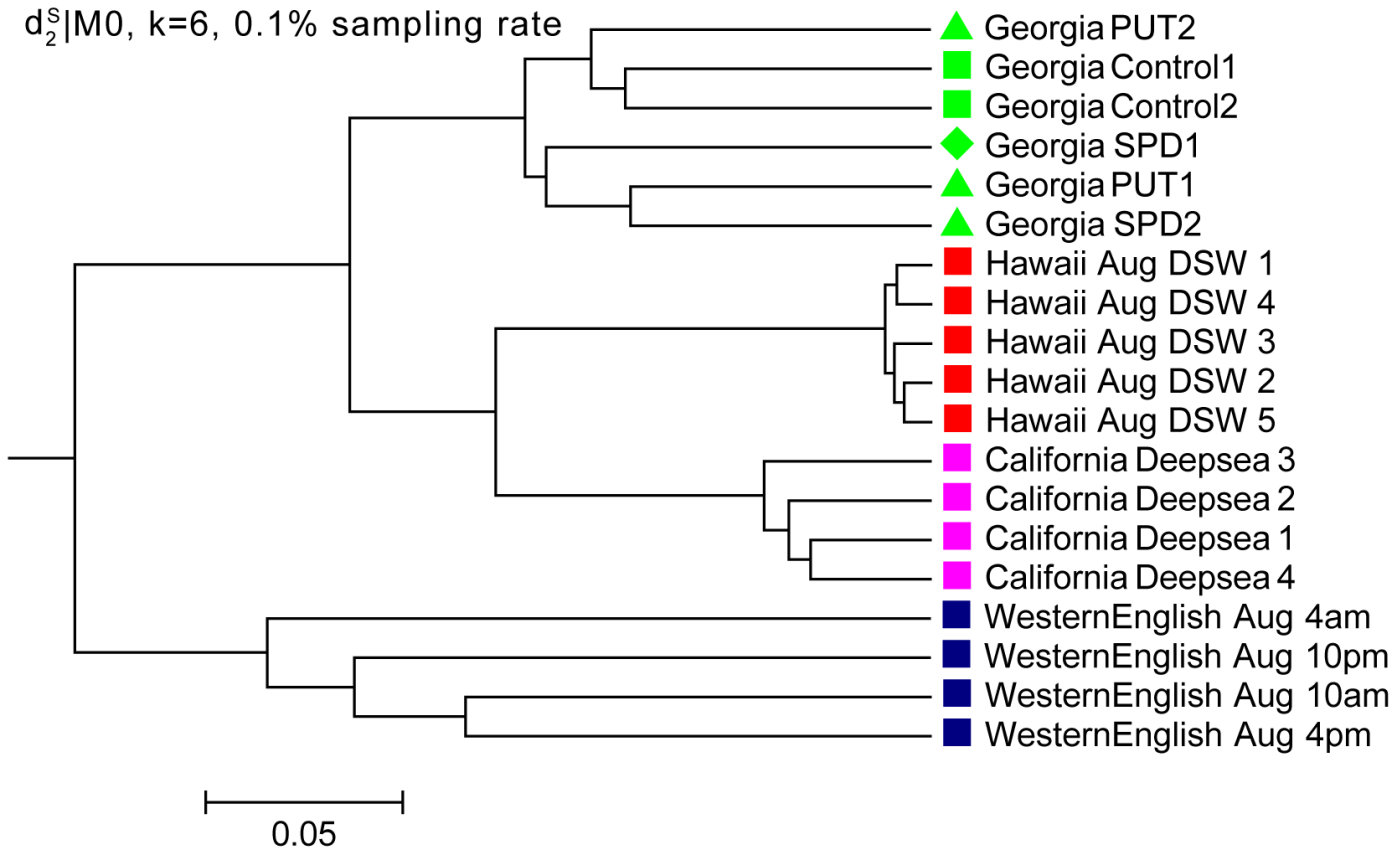
Clustering results of four communities under 0.1% sampling rate based on *d*_2_^*s*^|M_0_ and k = 6 in Experiment 1. *d*_2_^*s*^|M_0_ indicates using dissimilarity measure based on 0-th order Markov chain model. *d*_2_^*s*^can still cluster the four basic communities correctly, but cannot distinguish the subgroups in the Georgia community well.

To evaluate the effect of sequence depth on the performance of the different dissimilarity measures, we randomly sample 10%, 1% and 0.1% of the original reads from the 19 metatranscriptomic datasets. The read numbers are shown in Table S1 in Supplement S1. For 0.1% sampling rate, the minimum read number of the samples is only 86. We repeat the sampling experiments 100 times. The average symmetric difference scores between the clustering and the reference cluster with different tuple size *k* and dissimilarity measures are shown in Figure 4 and the detail scores are given in Tables S2, S3 and S4 in Supplement S1. For 10% sampling rate, the optimal score is 12,the same as that for the complete dataset. It shows that even under one-tenth sequencing depth, the 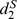 can still obtain the same satisfactory results as with the complete dataset. The performance of 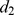 and 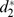 deteriorates compared with their performance with complete data, which indicates that their performances are significantly affected by the sequencing depth. However, for 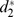, the clustering results are affected by the order of Markov model. The scores close to optimal can only be obtained under the 2^nd^ order and 3^rd^ order Markov model. For 1% sampling rate, the optimal average score is increased to about 14, and all measures cannot achieve the results as good as that using the complete data. When sampling rate drops to 0.1%, the 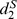 can still cluster the four basic communities correctly, but cannot distinguish the subclasses in the Georgia May community well, and the resulting clustering tree is shown in Figure 5. The performance of *Hao* deteriorates significantly when the tuple size increases. This trend is more obvious with the decrease of sampling rate, that is, when the sequencing depth is low. It might be caused by *Hao*’s attributions of the high number of parameters that need to be estimated to fit a Markov model of order *k*−2. *Ma* can achieve pretty good symmetric difference scores when the size *k* are between 8 to 10 at all sampling rates, which may attribute to its summing up the difference between two communities for all the 4*^k^ k*-tuples, which can reduce the bias from inefficient coverage when the sequencing depth is low. Other measures, such as *Ch*, *Eu*, and *S2,* do not perform well in all cases. It can be seen from Figure 4 that 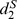 outperformed all the other measures under most situations.

**Figure 6:**
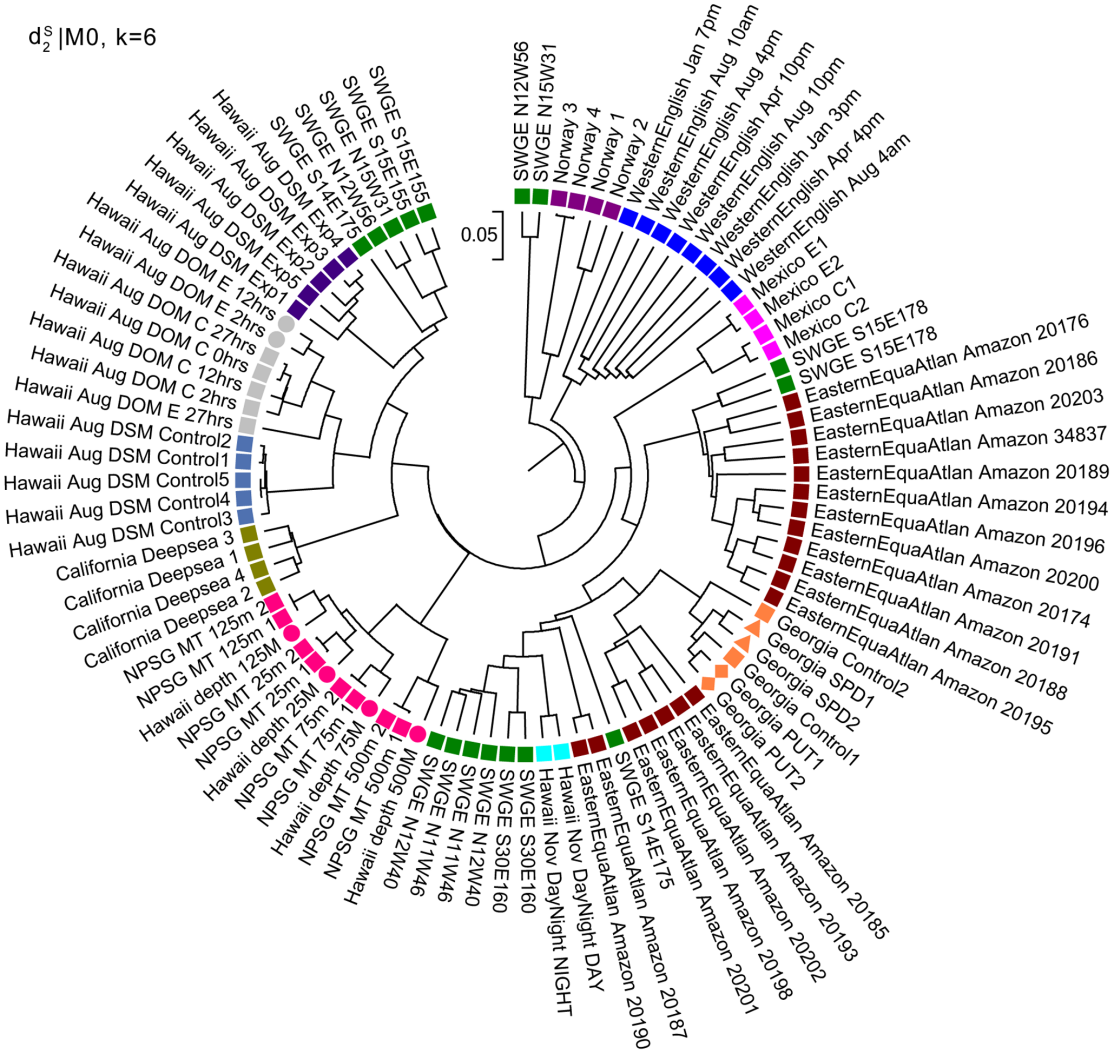
Clustering results of the 92 datasets from 12 communities based on *d*_2_^*s*^ |M_0_ and k = 6 in Experiment 1. *d*_2_^*s*^|M_0_ indicates using dissimilarity measure *d*_2_^*s*^ based on 0-th order Markov chain model. The dissimilarity measure *d*_2_^*s*^ can cluster most basic communities and subgroup control and amended samples correctly, validating the effectiveness of *d*_2_^*s*^.

For the outstanding performance of 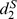 in all sequencing depth, 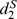 is applied to cluster all the 92 marine metatranscriptomic datasets from 12 projects with *k* = 6, and the simplest 0-th order Markov model. The clustering result is shown in Figure 6. Most samples from the same community are clustered together, except the Dataset 10 SWGE, which were collected from different locations of Equatorial North Atlantic Ocean and South Pacific Subtropical Gyre. Therefore, it is reasonable to see their samples clustered with different communities. Moreover, Dataset 9 (Hawaii_depth) and Dataset 12 (NPSG) were actually collected from the same location, ALOHA site, but using different preprocessing procedures before sequencing. The two datasets are merged first according to four different collecting depths, and then clustered together. This result validates the effectiveness and accuracy of 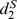 in samples clustering. It also reflects that the 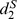 is robust to different sequencing platforms for dissimilarity measurements. We also observe that the clustering have obvious geographical tendency. The samples collected in Hawaii or close to Hawaii are clustered first. The samples from Norway and West English Channel, which are north latitudes in geographical location, are closely clustered. Most control samples and amended samples collected from the same locations are merged first respectively. For example, Mexico E1 and E2; Mexico C1 and C2 have a very clear hierarchical clustering order, and also the control samples and amended samples from Hawaii_Aug_DSM community. For Georgia community, except for the control samples, the SPD and PUT amended samples are clustered first respectively. For EasternEquaAtlan_Amazon community, the samples are merged into two main clusters which are very close to each other, which can be explained by the fact that the samples were collected during an oceanographic research cruise across the Amazon River plume to the eastern Equatorial Atlantic Ocean.

### Experiment 2: The Performance of Different Dissimilarity Measures in Recovering Gradient Relationships of Metatranscriptomic Samples

The gene expression levels in microbial communities may be controlled by a gradient such as ocean depth, temperature, and pH levels. In order to see the performance of the different dissimilarity measures in recovering the gradient relationships of the microbial communities, we used eight metatranscriptomic samples from 25 m, 75 m, 125 m and 500 m depth (two samples for each depth) of North Pacific Subtropical Gyre (NPSG) in ALOHA stations (datasets 12 on Table 1), which were sequenced with the pyrosequencing 454 platform. Except for the collection depth, other factors were the same for the eight communities.

Based on the *k-*tuple frequency vectors, the 16 dissimilarity measures are applied to calculate the dissimilarity between any pair of the eight samples. PCoA is then applied to assign for each sample a location in a low-dimensional space based on the dissimilarity matrix. The GOF value is the proportion of variance explained by the first principal coordinate and indicates the reliability of using the first principal coordinate to represent the data. Since the major difference of the eight samples of interest is collection depth, we expect that the principal coordinate can explain most of the differences of the samples and thus the GOF value is relatively high. The GOF of the first principal coordinates under each measure was shown in Table 3. From the table, we can see that, except for *S2* and *Ma*, the GOF by the first principal coordinate under most measures is higher than 0.70, indicating that the first principal coordinate can represent the data reasonably well. For a good dissimilarity measure, we expect that the first principal coordinate is highly associated with the collection depth. Therefore we calculate the SRCC between the first principal coordinate with the collection depth and the results are shown in Table 4. The highest SRCC 0.9759 is obtained under the 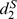 measure when k = 10 and 2^nd^ order Markov model indicating very high correlation between the first principal coordinate and the collection depth. Note that the corresponding GOF by the first principal coordinate is 0.77 indicating that the first principal coordinate represents the sample data well.

**Table 3:** The goodness of fit (GOF) value (times 100) by the first principal coordinate of PCoA in Experiment 2.

*k*	2	3	4	5	6	7	8	9	10
*d*_2_	80	79	79	79	80	81	82	82	82
*d*_2_^*s*^|M_0_	85	84	77	75	77	79	81	81	77
*d*_2_^*s*^|M_1_	69	71	77	75	77	78	79	79	75
*d*_2_^*s*^|M_2_	NA	40	81	78	78	78	79	79	75
*d*_2_^*s*^|M_3_	NA	NA	60	75	81	80	80	79	75
*d*_2_*|M_0_	82	82	79	74	70	68	63	59	56
*d*_2_*|M_1_	75	69	77	79	81	82	82	82	82
*d*_2_*|M_2_	NA	70	82	83	83	83	83	83	84
*d*_2_*|M_3_	NA	NA	53	82	82	84	86	88	90
*S2*|M_0_	69	59	56	40	17	15	14	14	14
*S2*|M_1_	73	78	84	83	34	17	15	14	14
*S2*|M_2_	NA	88	89	92	75	39	30	29	29
*Hao*	NA	62	80	80	85	89	88	74	42
*Ma*	78	65	60	62	66	68	68	67	63
*Eu*	84	84	90	94	95	96	96	96	95
*Ch*	98	98	99	99	99	99	99	99	99

The values on the table are the goodness of fit for the first principal coordinate times 100 for different tuple sizes and dissimilarity measures.

**Table 4:** The SRCC of the first principal coordinate and collection depth of the samples in Experiment 2.

*k*	2	3	4	5	6	7	8	9	10
*d*_2_	0.7807	0.6831	0.3904	0.3904	0.3904	0.4880	0.7807	0.7807	0.7807
*d*_2_^*s*^|M_0_	0.3904	0.3904	0.7807	0.7807	0.7807	0.7807	**0.8295**	0.7807	**0.9759**
*d*_2_^*s*^|M_1_	0.5855	0.5854	0.7807	0.7807	0.7807	0.7807	**0.8295**	0.7807	0.7807
*d*_2_^*s*^|M_2_	NA	0.5855	0.7807	0.7807	0.7807	0.7807	0.7807	0.7807	0.7807
*d*_2_^*s*^|M_3_	NA	NA	0.6831	0.7807	0.7807	0.7807	0.7807	0.7807	0.7807
*d*_2_*|M_0_	0.3904	0.3904	0.3904	0.3904	0.3904	0.3904	0.3904	0.0976	0.1952
*d*_2_*|M_1_	0.5855	0.7807	0.7807	0.7807	0.7807	0.7807	0.7807	0.7807	0.7807
*d*_2_*|M_2_	NA	0.2928	0.7807	0.7807	0.7807	0.7807	0.7807	0.7807	0.7807
*d*_2_*|M_3_	NA	NA	0	0.7807	0.7807	0.7807	0.7807	0.7807	0.7807
*S2*|M_0_	0.3904	0.1952	0.2440	0.3904	0.3904	0.4880	0.6831	0.7807	0.7807
*S2*|M_1_	0.1952	0.1952	0.3904	0.3904	0.3904	0.3904	0.3904	0.3904	0.3904
*S2*|M_2_	NA	0.1952	0.0976	0.2440	0.3904	0.3904	0.3904	0.3904	0.2440
*Hao*	NA	0.7807	0.7807	0.7807	0.7807	0.7807	0.7807	0.8783	0.0976
*Ma*	0.7807	0.7807	0.7807	0.7807	0.7807	0.7807	0.7807	0.7807	0.7807
*Eu*	0.7807	0.6831	0.3904	0.3904	0.3904	0.3904	0.3904	0.7807	0.3904
*Ch*	0.7807	0.4880	0.3904	0.3904	0.3904	0.3904	0.3904	0.3904	0.5367

The values on the table are Spearman’s Ranking Correlation Coefficients between the first principal coordinate and the collection depth of the samples for different tuple sizes and dissimilarity measures.

**Figure 7:**
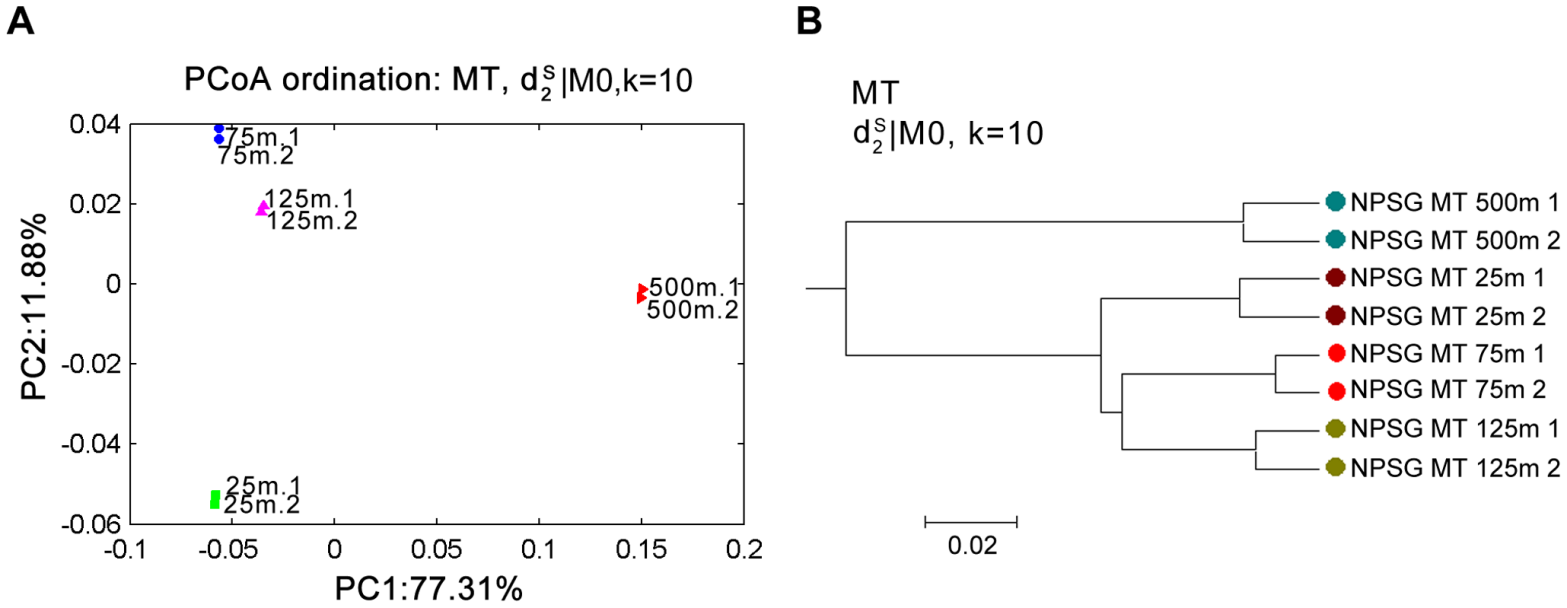
The PCoA ordinates of the NPSG data are primarily driven by the collection depth in Experiment 2. (A) is the two dimensional PCoA plots of the samples based on the dissimilarity measure *d*_2_^*s*^ for 0-th order Markov model and *k* = 10 (setting with highest SRCC); (B) is the clustering tree with *d*_2_^*s*^ for 0-th order Markov model and *k* = 10.

The two-dimension PCoA ordinates plot and the corresponding clustering results based on the dissimilarity matrix using the 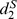 measure with tuple size k = 6 and 2nd order Markov model are shown in Figure 7. It can be seen from the figure that samples from the same depth are very close while samples from different depths are distant in the graph. The first principal coordinate explains 77% of variance among the eight samples. The zones of 25 m, 75 m and 125 m under the ocean belong to the photic zone and 500 m under the ocean belongs to the mesopelagic zone. Hence, the samples from 25 m, 75 m and 125 m areas under the ocean are clustered first, and the samples from 500 m are merged last, which is reasonable from the biological standpoint of view. The 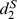 identified the depth-gradient variance better than other measures. For 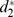, with 0-th order Markov model, the performance for all tuple sizes is poor. While with first order Markov model, the performance is significantly improved, which means that the order of Markov model has a large effect on the performance of the 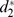 measure. This tendency is consistent with the observation in Experiment 1. For almost all other measures, the highest SRCC is 0.78, which means these measures can identify the gradient variance to some extent. For 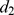, the performance is good when *k* is at least 8. The performance of *Hao* is reasonably good for *k* between 3 and 9, but deteriorates rapidly when *k* = 10. The relative performance of *Hao* with respect to tuple size *k* is consistent with that in Experiment 1. Similar to the results in Experiment 1, the performance of *Eu* and *Ch* is poor, while the performance of *Ma* is reasonable in recovering the gradient relationship between samples.

To see the effect of sequencing depth on the performance of the various dissimilarity measures in recovering gradient relationships of the microbial communities, we sample the eight metatranscriptomic datasets from four depths with 10%, 1% and 0.1% rates. The read numbers are shown in Table S5 in Supplement S1. At 0.1% sampling rate, the minimum read number of the samples is only 43. For each sampling rate, the random sampling is repeated 100 times, and the average GOF values by the first principal coordinate at each sampling rate are shown in Table S6, S7, and S8 in Supplement S1. From Table S6, except for the dissimilarity measures *S2* and *Ma* and for large tuple size of *k* = 10, the GOF values are all above 0.5. The average SRCCs are shown in Table S9 in Supplement S1. For 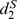, with 74% GOF, the optimal SRCC is 0.98, the same as that with complete data, which means 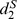 still maintains good performance using 10% of the reads. The other dissimilarity measures also yield similar performance using 10% of the data as with complete data, but do not perform better than 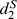. At 1% and 0.1% sampling rates, most GOF values are much smaller than that obtained with the complete data. With the increase of tuple size and the order of Markov model, the GOF values decrease dramatically. So the first principal coordinate does not explain the differences among the communities well. Thus, the SRCC analysis between the principal coordinate and the collection depth is not highly meaningful.

### Experiment 3: Using the Dissimilarity measures to Cluster Metagenomic and Metatranscriptomic Datasets

**Figure 8:**
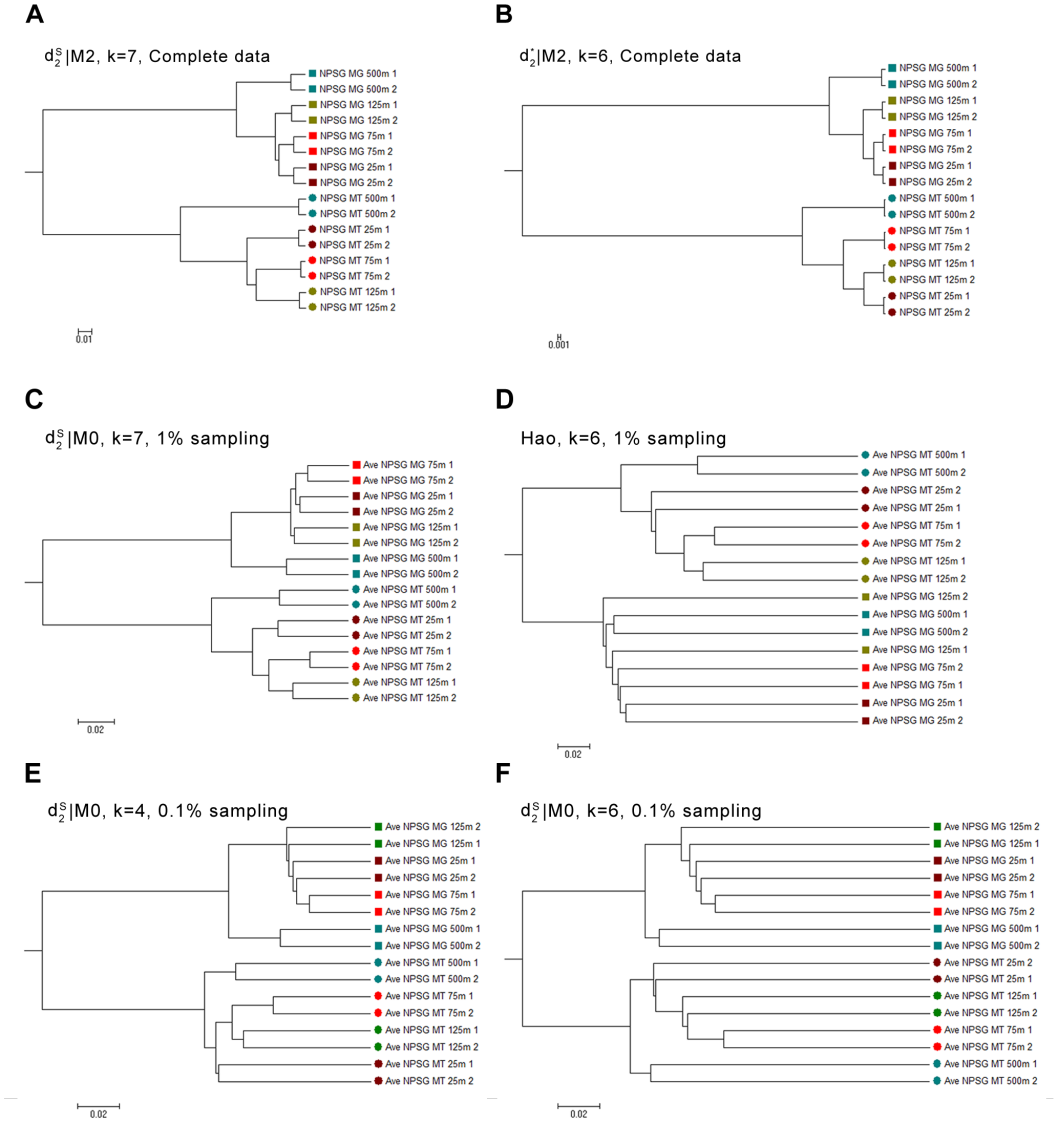
Clustering results of the metagenomic and metatranscriptomic datasets from the NPSG community in Experiment 3. (A) (B) are the clustering results using *d*_2_^*s*^ the complete data under *d*_2_^*s*^ with k = 7 and *d*_2_* with *k* = 6 under 2^nd^ order Markov chain model; (C) and (D) are the clustering results based on average dissimilarity using *d*_2_^*s*^ with k = 7 under 0-th order Markov chain model and *Hao* with *k* = 6 based on 100 times of 1% sampling from the original data; (E) and (F) are the clustering results based on average dissimilarity using *d*_2_^*s*^ when *k* = 4 and *k* = 6 for the 0-th order Markov chain based on 100 times of 0.1% sampling from the original data.

We next used the dissimilarity measures to cluster metagenomic and metatranscriptomic samples. Our objective is to see if metagenomic samples and metatranscriptomic samples separate into two groups. The samples from collection depth of 25 m, 75 m, 125 m and 500 m (two samples for each depth) of North Pacific Subtropical Gyre (NPSG) in ALOHA stations (Dataset 12 on Table 1) were sequenced as eight metagenomic and eight metatranscriptomic datasets with the pyrosequencing 454 platform. The dissimilarity measures based on sequence signatures are applied to measure the beta diversity between these datasets. The clustering results based on all the measures show obvious tendency that the metagenomic and metatranscriptomic datasets are clustered into two separate groups. However, the measures *Ch*, *Eu* and *Hao* for large tuple size *k* cannot completely distinguish the two types of data with some metatranscriptomic and metagenomic datasets mixed together, which are shown in the Figure S1 in Supplement S1. The observation of poor performance of *Hao* for large tuple size validates the analysis in Experiments 1 and 2. Except for *k* = 2 with 1^st^ order Markov model for 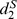 and 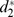, and small tuple size for 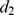 and *Ma*, all the other settings under 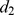, 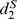, 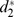 and *Ma* can distinguish the metagenomic and metatranscriptomic datasets indicating metagenomic and metatranscriptomic data have different sequence signature information. However, the hierarchical clustering of sub-classes within either the metagenomic or the metatranscriptomic samples shows the performance differences of these measures. As shown in Figure 8 (A) and (B), for 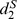, the samples from adjacent depths are merged first (25 m & 75 m, or 75 m & 125 m); while for all the other measures, such as 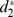, from *k* = 4–10 with 2^nd^ order Markov model, the samples from non-adjacent depths (125 m and 25 m in MT) are merged first, which is not reasonable based on basic biological knowledge. Therefore, the 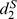 measure shows outstanding performance when the metatranscriptomic and metagenomic datasets co-exist. Most measures have the tendency to distinguish the metatranscriptomic and metagenomic datasets, but show performance difference in subclass details.

The eight metatranscriptomic and eight metagenomic datasets from four depths are also randomly sampled with 10%, 1% and 0.1% rates to evaluate the performance of the different dissimilarity measures when the sequencing depth is low. The read numbers are shown in Table S10 in Supplement S1. For 0.1% sampling rate, the minimum read number of the samples is only 43. The sampling rate affects the sub-classes within the metagenomic and metatranscriptomic datasets. However, the basis grouping of metagenomic and metatranscriptomic data can still be well distinguished even under the 0.1% sampling rate. With 1% sampling rate, 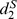 still yields the same satisfactory results as that with complete data when *k* = 6, as shown on figure 8(C). While for *Hao*, the performance drops dramatically with low sequencing depth, even the tuple size is medium (k = 6), shown on figure 8(D). When the sampling rate is 0.1%, even the 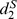 cannot achieve as good results as using the complete data. The clustering results are also sensitive to tuple size, as shown on figure 8(EF). For 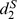, when *k* = 4, the clustering in metatranscriptomic dataset are still correct, while when the tuple size increases from 4 to 6, the clustering results become worse.

**Figure 9:**
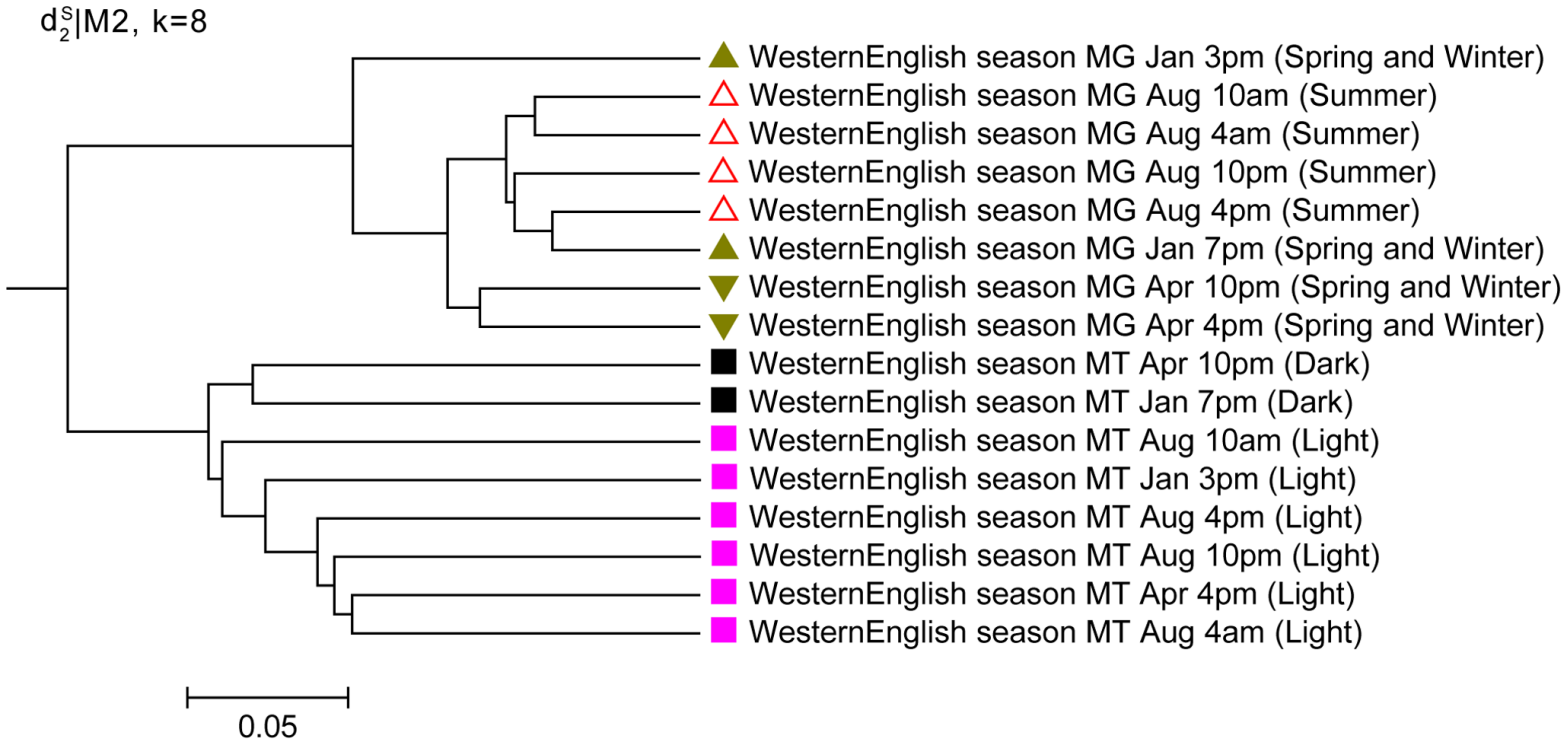
Clustering results of the Western English Channel based on *d*_2_^*s*^|M_2_ and *k* = 8 in Experiment 3. The datasets contains the metagenomic and metatranscriptomic samples collected from different times. *d*_2_^*s*^|M_2_ indicates using dissimilarity measure *d*_2_^*s*^based on 2^nd^ order Markov chain model. As shown in the parentheses after each data, there are clear diurnal variation and season patterns for MT and MG clustering, respectively.

We also analyze eight metagenomic and eight metatranscriptomic samples from the Western English Channel sequenced with pyrosequencing 454 platform [9] (Dataset11 in table 1). We investigate how well the dissimilarity measures cluster these samples. Similarly, the metagenomic and metatranscriptomic datasets are clearly clustered into two groups further validating that the sequence signatures are different for metagenomic and metatranscriptomic datasets. As shown in Figure 9, the clustering of metatranscriptomic data from marine presents a clear pattern, where 4 am of Aug.(dawning), 4 pm of April(dusk), 3 pm of Jan.(dusk) and 10 pm of Aug.(dusk); 10 am of Aug. and 4 pm of Aug.(both daytime) are clustered together, while 10 pm of April and 7 pm of Jan. (both dark) are cluster together. This means that photosynthesis might play an important role for the gene expression of microbes. Therefore, 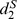 can reveal the differential expression pattern among the metatranscriptomic datasets. At the same time, the metagenomic data from the same location are clustered with a season pattern, where the data collected in Aug. are together and those from Jan. and April are together.

### Experiment 4: The Performance of Different Dissimilarity Measures using Sequence Signatures for Clustering Metatranscriptomic Datasets from the Illumina Sequencing Platform

Seven metatranscriptomic datasets collected from cecum and colon tissues of four mice were sequenced with the Ilumina Genome Analyzer IIx (GaIIx) platform and they were downloaded from NCBI SRA. The description can be found in Dataset13 of Table 1.

**Figure 10:**
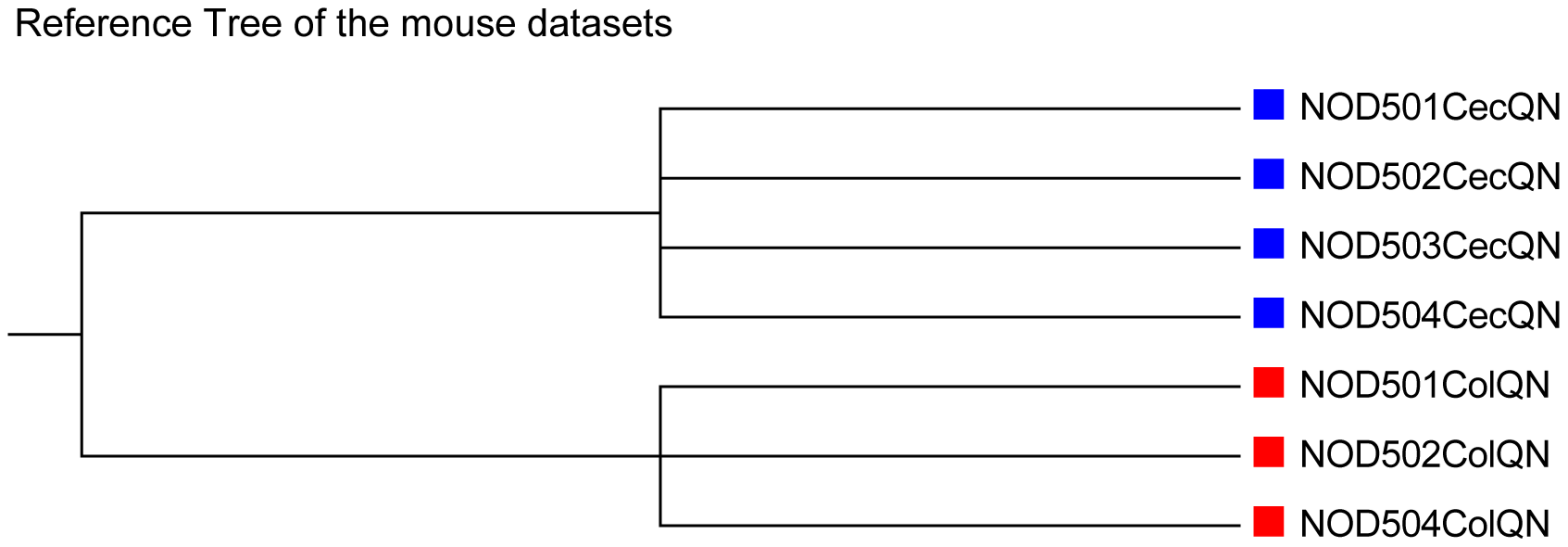
The reference tree of the mouse datasets in Experiment 4. The seven samples are clustered according to their tissue types. The sample ID, such as ‘NOD504ColQN’, where the digital numbers, ‘504’, are the mouse ID, ‘Cec’ means cecum and ‘Col’ means colon, ‘QN’ means Qiagen-based protocol.

According to the two tissues the datasets coming from, the seven samples can be clustered into two distinct groups, as shown in Figure 10, without distance information included. Based on the *k-*tuple frequency vectors and the 16 dissimilarity measures, the dissimilarity between each sample-pair is obtained. With the dissimilarity matrix, hierarchical clustering is implemented with UPGMA. The symmetric differences between the clustering results and reference cluster under various measures, tuple size and order of Markov models are calculated as shown in Table 5.

**Table 5:** Symmetric differences between clustering and reference tree in Experiment 4.

*k*	2	3	4	5	6	7	8	9	10
*d*_2_	7	7	7	7	7	7	7	7	7
*d*_2_^*s*^|M_0_	7	7	***5***	***5***	***5***	***5***	***5***	***5***	***5***
*d*_2_^*s*^|M_1_	7	7	7	7	***5***	***5***	***5***	***5***	***5***
*d*_2_^*s*^|M_2_	NA	7	7	7	7	***5***	***5***	***5***	***5***
*d*_2_^*s*^|M_3_	NA	NA	7	7	7	7	***5***	***5***	***5***
*d*_2_*|M_0_	7	7	7	7	7	7	7	7	7
*d*_2_*|M_1_	7	7	7	7	7	7	7	7	7
*d*_2_*|M_2_	NA	7	7	7	7	7	7	7	7
*d*_2_*|M_3_	NA	NA	7	7	7	7	7	7	7
*S2*|M_0_	7	7	7	7	***5***	***5***	***5***	7	7
*S2*|M_1_	7	7	7	7	***5***	***5***	***5***	7	7
*S2*|M_2_	NA	7	7	7	***5***	***5***	***5***	***5***	7
*Hao*	NA	7	7	7	***5***	***5***	***5***	***5***	***5***
*Ma*	7	7	7	***5***	***5***	***5***	***5***	***5***	***5***
*Eu*	7	7	7	7	7	7	7	7	7
*Ch*	7	7	7	7	7	7	7	7	7

score = 5, p-value = 0.043; score = 7, p-value = 1.0.

**Figure 11:**
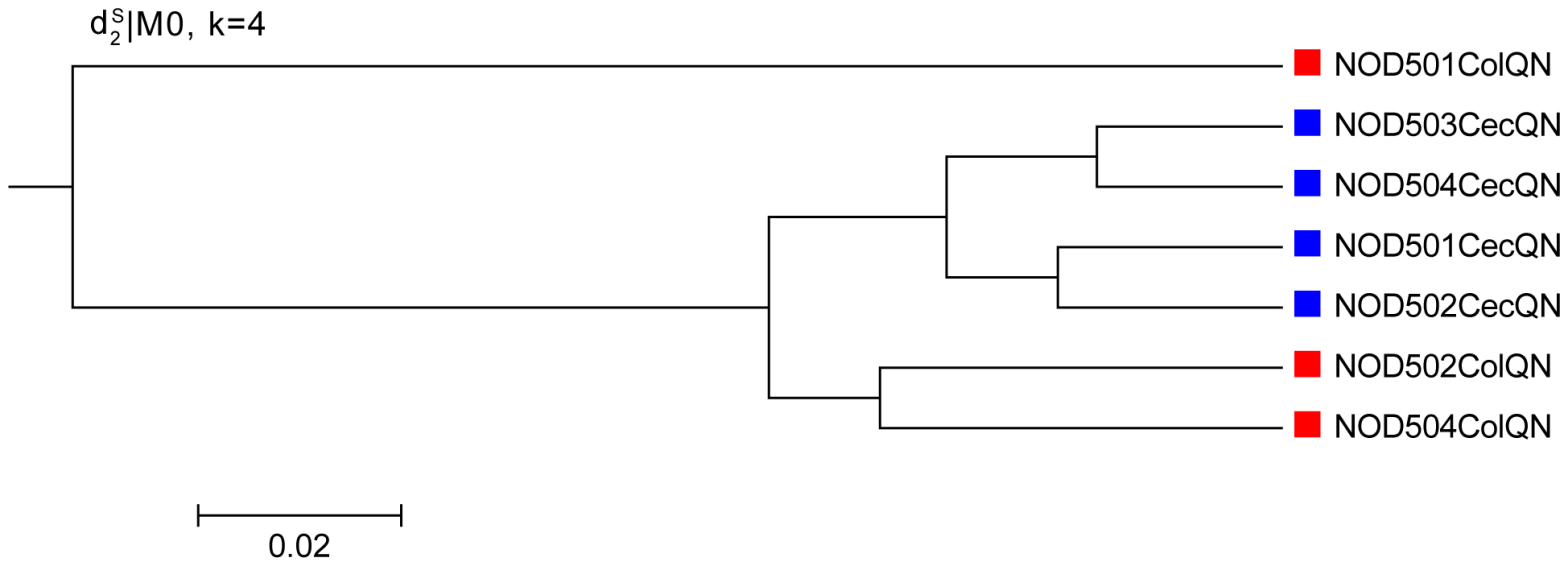
Clustering results of the mouse datasets based on *d*_2_^*s*^|M_0_ and k = 4 in Experiment 4. *d*_2_^*s*^|M_0_ indicates using dissimilarity measure *d*_2_^*s*^ based on 0-th order Markov chain model. Clusters for the four cecum samples are correct. For the three colon samples, two of them are clustered correctly, while the other one is merged at last.

In Table 5, the optimal symmetric difference score between the reference tree and clustering results is 5. 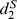, *S2*, *Hao* and and *Ma* can achieve this optimum score with appropriate choices of tuple size *k*. However, the range of the values *k* that yields the optimal results is different. For 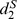, the optimal score is obtained for k = 4 to 10 for the 0-th order Markov chain and for relatively narrow ranges of *k* with higher orders of Markov chain. For *S2*, the optimal results are obtained when *k* is between 6–8 with the order of the Markov chain playing no roles. For *Hao* and *Ma*, the optimal results are obtained for *k* between 6–10. One of the optimal clustering trees with k = 4 and 0-th order Markov model under 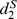 is shown in Figure 11. The four samples from cecum are clustered together, and two of the colon samples are clustered together, while the remaining colon sample is finally merged. The results show that the sequence signature measures are valid for sequencing data from the Illumina platform and 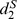 still keeps the outstanding performance for all orders of Markov chains.

**Figure 12:**
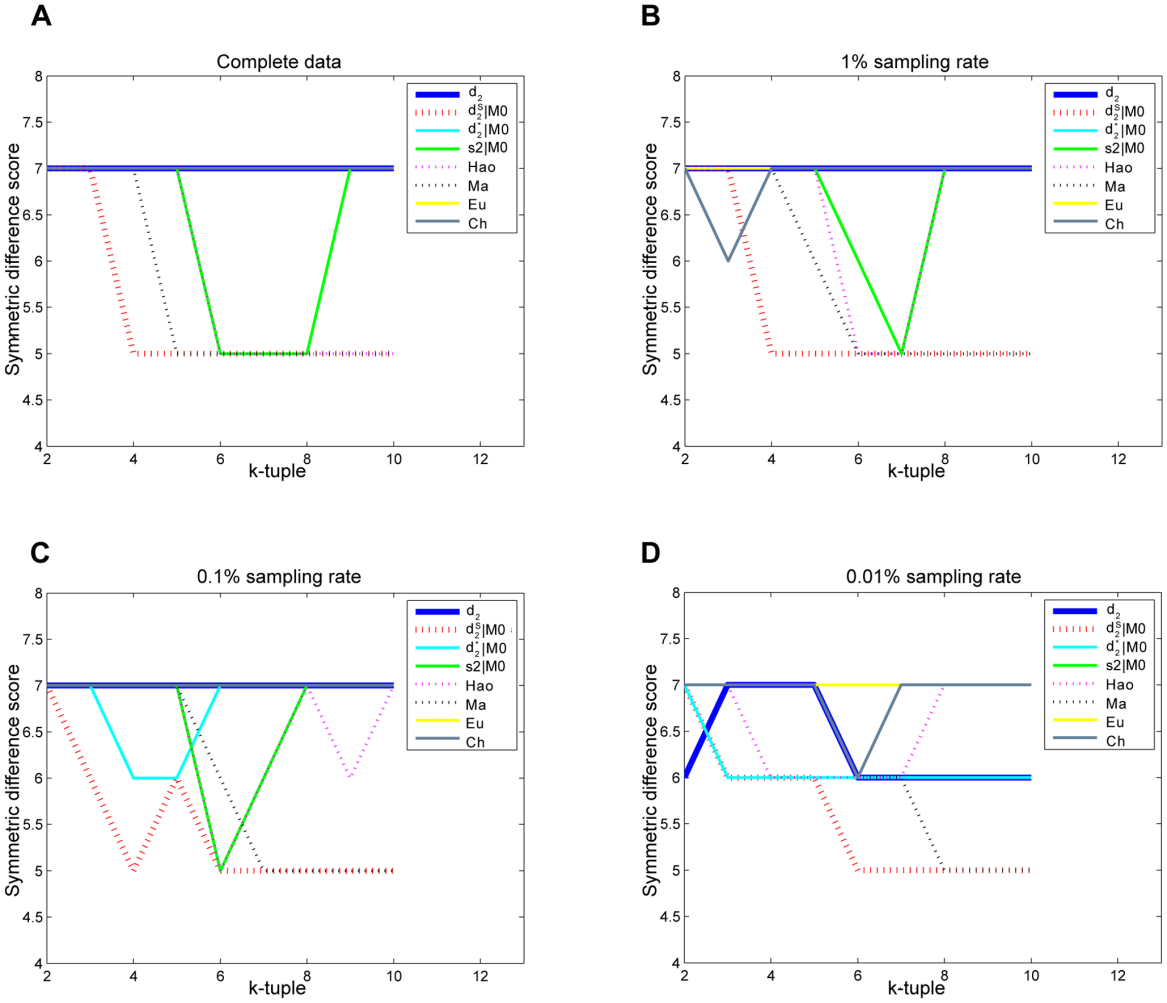
Average symmetric difference scores for the mouse datasets under different sampling rates in Experiment 4. (A) is the symmetric difference scores as a function of tuple size *k* for different dissimilarity measures based on the complete data. (B), (C) and (D) are the average symmetric difference scores as a function of tuple size *k* for different dissimilarity measures based on 100 random samplings of 1%, 0.1% and 0.01% sampling rates, respectively. The lower the score is, the closer the clustering results and reference tree is. It is clear that *d*_2_^*s*^ shows best performance under most of the conditions.

To evaluate the effect of sequence depth on the measures’ performance with Illumina datasets, we randomly sample 1%, 0.1% and 0.01% of the original reads from the seven metatranscriptomic datasets. The reason we sampled with 10% sampling rate for 454, but not for Illumina, is due to the high sequencing depth of Illumina data. The read numbers are shown in Table S11 in Supplement S1. For 0.01% sampling rate, the minimum read number of the samples is only 115. We repeat the sampling experiments 100 times. The average symmetric difference scores between the clustering and the reference cluster with different tuple size *k* and dissimilarity measures are shown in Figure 12 and the detail scores are given in Tables S12, S13 and S14 in Supplement S1. For 1% and 0.1% sampling rate, the 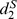 can still obtain the same optimal results under the same range of *k* value as the complete dataset. However, for 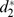 and *Hao*, althougth they can still obtain the optimal results, the range of optimal *k* value become smaller compared with the complete datasets. The results show that compared with other measures, 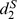 is robust to sequencing depth. The performance of *Ma* deteriorates slightly with the decrease of sampling rate, which can obtain the optimal clustering results with slightly narrower range of tuple size *k.* However, the performances of other measures, including *Hao*, *Ma* and *S2,* become worse when coverage decreases, and except the *Ma*, other measures cannot obtain the optimal clustering results, as shown on Table S14 in Supplement S1. In this study, 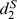 shows its outstanding performance for datasets from the Illumina sequencing platform.

### Experiment 5: The Effects of Sequencing Errors on the Performance of Different Dissimilarity Measures

The 19 metatranscriptomic samples of 4 communities having distinct geographic locations used in Experiment 1 are used to study the effect of sequencing errors on the performance of sequencing signature measures. According to sequencing error features of the 454 platform [37]–[39], we added 1% indel errors and 0.1% substitution errors to the original sequencing data. The simulated reads set with the preset error rates are generated with a pyrosequencing 454 simulator, FlowSim [37]. The same clustering procedures as in Experiment 1 are implemented. The symmetric differences and the comparison with the results based on the original data are shown in Table 6. The clustering results do not change much under 1% indel and 0.1% substitution error rates. For 0–3 orders of Markov chain of 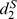 and 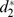, only 2 among 36 values are changed for each measure compared with the results based on the original data. *Ma* keeps the same symmetric differences as the original data. Many more of the symmetric differences, 3–7 among 9 values, for 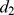, S2, *Hao*, *Eu* and *Ch*, are changed.

**Table 6:** Symmetric differences between clustering and reference tree under 1% indel errors and 0.1% substitution errors in Experiment 5 and comparison with Table 2 in Experiment 1.

	Symmetric differences									Variation of symmetric differences between original and imported-error data (Experiment 5 - Experiment 1)								
*K*	2	3	4	5	6	7	8	9	10	2	3	4	5	6	7	8	9	10
*d*_2_	14	12	14	14	14	14	12	12	12	−2	−4	−2	−2	0	0	0	0	0
*d*_2_^*s*^|M_0_	18	14	16	16	12	12	12	14	14	0	0	0	0	0	0	0	0	0
*d*_2_^*s*^|M_1_	14	16	16	16	12	12	12	12	14	0	0	0	0	0	0	0	0	0
*d*_2_^*s*^|M_2_	NA	14	16	16	12	12	12	14	14	NA	0	0	0	0	0	0	2	0
*d*_2_^*s*^|M_3_	NA	NA	14	16	12	12	12	12	12	NA	NA	0	0	0	0	0	0	−2
*d*_2_*|M_0_	18	14	16	16	14	12	12	12	12	0	0	0	0	2	0	0	0	0
*d*_2_*|M_1_	16	14	16	16	12	12	12	12	12	2	0	0	0	0	0	0	0	0
*d*_2_*|M_2_	NA	14	16	16	12	12	12	12	12	NA	0	0	0	0	0	0	0	0
*d*_2_*|M_3_	NA	NA	14	12	12	12	12	12	12	NA	NA	0	0	0	0	0	0	0
*S2*|M_0_	22	22	18	18	18	18	16	14	22	0	0	0	0	0	0	−2	−4	6
*S2*|M_1_	22	20	18	18	18	18	18	14	22	0	0	0	0	0	0	0	−4	6
*S2*|M_2_	NA	18	18	18	18	18	16	14	22	NA	0	0	0	0	0	−2	−4	6
*Hao*	NA	14	16	16	12	12	12	12	12	NA	0	0	0	−2	−2	0	0	−2
*Ma*	16	16	16	18	18	12	12	14	14	0	0	0	0	0	0	0	0	0
*Eu*	16	14	18	16	16	16	16	18	18	0	−2	0	−2	0	0	0	4	4
*Ch*	14	16	20	22	18	22	20	22	22	−2	−6	2	0	−2	2	2	2	0

For all scores, p-value<0.001. The columns are the values for tuple size from 2 to 10. The first big column are the symmetric difference values for the tuple size. The second big column is the absolute value of variation of symmetric differences between original and imported-error data.

If a measure can obtain optimal clustering results based on the original data, it keeps the performance on imported-error data. If the measure cannot obtain the optimal results with the original data, the measure will not achieve the best performance either with added errors. Therefore, the relative performances of the dissimilarity measures are not affected by the sequencing error rates for the current sequencing technologies.

## Discussion and Conclusions

In this study, we evaluated the performance of *k*-tuple based sequence signature methods to compare microbial metatranscriptomic samples with NGS reads data. We studied three 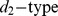 dissimilarity measures, one dissimilarity measure in CVTree, one relative entropy based measure and three classical 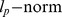 distances. These dissimilarity measures, together with tuple size *k* = 2–10 and different Markov background models, were compared on the basis of five experiments of real metatranscriptomic datasets from global marine communities, with the objectives to explore their performance on clustering metatranscriptomic sequencing data from different communities generated by pryosequencing 454 and Illumina platforms, identifying gradient variance of metatranscriptomic datasets, clustering characteristics when metagenomic and metatranscriptomic datasets co-exists and robustness under sequencing errors.

For geographically well separated communities, all the measures can classify the big groups correctly. With the complete data, for certain range of tuple size *k*, 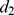, 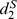, 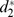, Hao and Ma can classify the subgroups and obtain the closest clustering results from the reference cluster. When sequencing depth is low, only 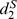 still keep the outstanding performance and other measures are more sensitive to sequencing depth. Even for the 92 samples from 12 communities, most measures can cluster major groups correctly and 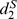 can merge the communities according to similar geographical locations. The *k*-tuple dissimilarity measures can reflect the gradient tendency, and 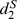 can obtain the highest correlation coefficient between the first principal coordinate and the gradient. When metagenomic and metatranscriptomic datasets co-exist, all the measures can cluster the samples according to the data type. We also evaluate the effects of tuple size and background model. In this study, tuple size *k = *2–10, and the performance of different dissimilarity measures varies with different tuple size. The order of background Markov model does not affect the performance significantly.

Our results indicate that 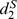 performs satisfactorily for grouping microbial communities, identifying their gradient relationships and separating metagenomic and metatranscriptomic communities. The 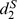 dissimilarity measure performs similarly in some scenarios or outperforms other dissimilarity measures in many other scenarios and its performance is not highly sensitive to tuple size, which makes it easier to apply to real data. It is a powerful approach for metatranscriptomic sample comparison based on NGS shotgun reads. For 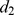, relationship between the sequences in both samples plays less effects than the variation of the tuple occurrences within one sample, which lead to its relative poor performance. *Hao*’s attributions of the high number of parameters that need to be estimated to fit a Markov model of order *k*−2 leads to the poor performance under low sequencing depth. *Ch* considers the maximum difference between the tuple frequencies for the samples only and does not make full use of the information from all the tuples. On the other hand, *Ma* sums up the difference between two communities for all the 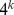 *k*-tuples, which can reduce the bias from low coverage when sequencing depth is low. The normalization of the tuple counts by their corresponding expectations plays an important role in the superior performance of 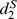 and 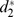.

The performance of different dissimilarity measures varies with the tuple size. We show that 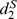 and 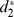 can achieve reasonable clustering results for metatranscriptomic datasets. In particular, the 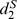 dissimilarity measure outperforms others in most scenarios and its performance is not highly sensitive to the tuple size. Thus, it is a powerful approach for metatranscriptomic sample comparison based on NGS shotgun reads. The dissimilarity measure 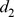 performs reasonably well when the tuple size is relatively high. However, it does not perform well when the tuple size is low. These observations are consistent with the results for the comparison of metagenomic datasets. The *Hao* dissimilarity measure performs reasonably well when sequence depth is high and the tuple size is relatively low. One explanation is that it compares the numbers of occurrences of *k*-tuples with their corresponding expectations based on the *k*−2 order of Markov chain, which may not be accurate especially when the sequence depth is low and the tuple size is high. *Ch* considers the maximum difference between the tuple frequencies of the samples only and does not make full use of the information from all the tuples. On the other hand, *Ma* sums up the differences of all the *k*-tuple frequencies between two communities, which can reduce the bias from inefficient coverage when the sequencing depth is low. The normalization of the tuple counts by their expectations plays an important role in the superior performance of 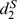 and 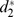.

The study design in this paper is similar to that in Jiang et al. [25]. The objective of this study is to see whether the conclusions about the relative performance of alignment-free methods for metagenomic comparison observed in Jiang et al. [25]are also true for the comparison of metatranscriptomes. This conclusion is not obvious due to the different characteristics of metatranscriptomic from metagenomic data. Previous study of the effectiveness of alignment-free approaches on metagenomic datasets is built on the theoretical basis [14] that *k*-tuple frequencies are similar across different regions of the same genome, but differ between genomes. However, in metatranscriptomic data, the genes within a genome can have different expression levels and the intron and inter-genetic region sequences are removed, while in metagenomic data, all the genomic regions are the same. At the same time, RNA the different characteristics from DNA, such as degradation, stability, easiness to be broken and alternative splicing, etc., which bring the different preferences and bias distributions to the sequencing process. Therefore, under the situation that: the expression abundance information is imported, the sequences of intron and inter-genic regions are taken out, and different sequencing preference and bias are introduced, whether the alignment-free approaches are valid is a critical question for their further applications to the metatranscriptomic datasets.

Similar conclusions about the relative performance of the different dissimilarity measures on clustering microbial metagenomic and/or metatranscriptomic communities were obtained. Our conclusion about the applicability of alignment-free statistics, in particular 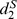, for the comparison of metatranscriptomic samples is of biological significance. In addition, we showed that metagenomic and metatranscriptomic samples can be separated by using alignment-free statistics as shown in Figure 9, which cannot be achieved by studying metagenomic or metatranscriptomic dataset alone. Since the sequencing data, sequencing platform, and the microbial communities from the two studies were widely different, we believe that 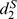 can be used to effectively cluster both metagenomic and metatranscriptomic communities. Therefore, *k*-tuple based sequence signature methods such as 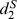 and 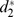 are simple and computationally efficient for the comparison of metatranscriptomic data, and they provide attractive alternative approaches for microbial community comparison.

## Supporting Information

Supplement S1 Figure S1. The clustering results of both metagenomic and metatranscriptomic datasets in Experiment 3 based on the dissimilarity measures *Ch*, *Hao* and *Eu*. Table S1. Sampling reads number for Experiment 1. Table S2. The symmetric difference between the reference and clustering trees for Experiment 1 (10% sampling). Table S3. The symmetric difference between the reference and clustering trees for Experiment 1 (1% sampling). Table S4. The symmetric difference between the reference and clustering trees for Experiment 1 (0.1% sampling. Table S5. Sampling reads number for Experiment 2. Table S6. The GOF (times 100) by first principle coordinate of PCoA of Experiment 2 (10% sampling). Table S7. The GOF (times 100) by first principle coordinate of PCoA of Experiment 2 (1% samping). Table S8. The GOF (times 100) by first principle coordinate of PCoA of Experiment 2 (0.1% sampling). Table S9. The SRCC between the first principle coordinate and depth for Experiment 2 (10% sampling). Table S10. Sampling reads number for Experiment 3. Table S11. The number of sampling reads for Experiment 4. Table S12. The symmetric difference between the reference and clustering trees in Experiment 4 (1% sampling). Table S13. The symmetric difference between the reference and clustering trees in Experiment 4 (0.1% sampling). Table S14. The symmetric difference between the reference and clustering trees in Experiment 4 (0.01% sampling).
(DOC)

## References

[1] Lozupone C, Lladser M, Knights D, Stombaugh J, Knight R (2007) UniFrac: an effective distance metric for microbial community comparison. ISME J 5: 169–172. 10.1038/ismej.2010.133 PMC3105689 20827291

[2] Smith T, Waterman M (1981) Comparison of biosequences. Adv Appl Math 2: 482–489.

[3] Altschul S, Gish W, Miller W, Myers E, Lipman Dea (1990) Basic local alignment search tool. J Mol Biol 215: 403–410. 2231712 10.1016/S0022-2836(05)80360-2

[4] Dick GJ, Andersson AF, Baker BJ, Simmons SL, Thomas BC, et al (2009) Community-wide analysis of microbial genome sequence signatures. Genome Biol 10: 85. 10.1186/gb-2009-10-8-r85 PMC2745766 19698104

[5] Dick GJ, Clement BG, Webb SM, Fodrie FJ, Bargar JR, et al (2009) Enzymatic microbial Mn oxidation in the Guaymas Basin deep-sea hydrothermal plume. Geochim Cosmochim Ac 73: 6517–6530.

[6] Dick GJ, Tebo BM (2010) Microbial diversity and biogeochemistry of the Guaymas Basin hydrothermal plume. Environ Microbiol Rep 12: 1334–1347. 10.1111/j.1462-2920.2010.02177.x 20192971

[7] Ghosh T, Mohammed M, Rajasingh H, Chadaram S, Mande S (2011) HabiSign: a novel approach for comparison of metagenomes and rapid identification of habitat-specific sequences. BMC Bioinformatics 12: 59–69. 22373355 10.1186/1471-2105-12-S13-S9 PMC3278849

[8] Gilbert JA, Field D, Swift P, Thomas S, Cummings D, et al (2010) The taxonomic and functional diversity of microbes at a temperate coastal site: a ‘multi-omic’ study of seasonal and diel temporal variation. PLoS ONE 5: e15545. 21124740 10.1371/journal.pone.0015545 PMC2993967

[9] Gilbert JA, Meyer F, Schriml L, Joint IR, Mühling M, et al (2010) Metagenomes and metatranscriptomes from the L4 long-term coastal monitoring station in the Western English Channel. Stand Genomic Sci 3: 183–193. 21304748 10.4056/sigs.1202536 PMC3035373

[10] Jay McCarrena, Jamie W Beckera, Daniel J Repetac, Yanmei Shia, Curtis R Younga, et al (2010) Microbial community transcriptomes reveal microbes and metabolic pathways associated with dissolved organic matter turnover in the sea. Proc Natl Acad Sci USA 107: 16420–16427. 20807744 10.1073/pnas.1010732107 PMC2944720

[11] Mou X, Vila-Costa M, Sun S, Zhao W, Sharma S, et al (2011) Metatranscriptomic signature of exogenous polyamine utilization by coastal bacterioplankton. Environ Microbiol Rep 3: 798–806. 23761372 10.1111/j.1758-2229.2011.00289.x

[12] Poretsky R, Hewson I, Sun S, Allen A, Zehr J, et al (2009) Comparative day/night metatranscriptomic analysis of microbial communities in the North Pacific subtropical gyre. Environ Microbiol 11: 1358–1375. 19207571 10.1111/j.1462-2920.2008.01863.x

[13] Shi Y, Tyson GW, Eppley JM, DeLong EF (2011) Integrated metatranscriptomic and metagenomic analyses of stratified microbial assemblages in the open ocean. ISME J 5: 999–1013. 21151004 10.1038/ismej.2010.189 PMC3131857

[14] Karlin S, Mrazek J, Campbell A (1997) Compositional biases of bacterial genomes and evolutionary implications. J Bacteriol 179: 3899–3913. 9190805 10.1128/jb.179.12.3899-3913.1997 PMC179198

[15] Blaisdell B (1986) A measure of the similarity of sets of sequences not requiring sequence alignment. Proc Natl Acad Sci USA 83: 5155–5159. 3460087 10.1073/pnas.83.14.5155 PMC323909

[16] Hide W, Burke J, DA Vison D (1994) Biological evaluation of d2, an algorithm for highperformance sequence comparison. J Comput Biol 1: 199–215. 8790465 10.1089/cmb.1994.1.199

[17] Miller R, Christoffels A, Gopalakrishnan C, Burke J, Ptitsyn A, et al (1999) A comprehensive approach to clustering of expressed human gene sequence: the sequence tag alignment and consensus knowledge base. Genome Res 9: 1143–1155. 10568754 10.1101/gr.9.11.1143 PMC310831

[18] Kantorovitz MR, Robinson GE, Sinha S (2007) A statistical method for alignment-free comparison of regulatory sequences. Bioinformatics 23: 249–255. 17646303 10.1093/bioinformatics/btm211

[19] Reinert G, Chew D, Sun FZ, Waterman MS (2009) Alignment-free sequence comparison (I):Statistics and power. J Comput Biol 16: 1615–1634. 20001252 10.1089/cmb.2009.0198 PMC2818754

[20] Wan L, Reinert G, Sun F, Waterman M (2010) Alignment-free sequence comparison (ii): theoretical power of comparison statistics. J Comput Biol 17: 1467–1490. 20973742 10.1089/cmb.2010.0056 PMC3123933

[21] Dai Q, Wang T (2008) Comparison study on k-word statistical measures for protein: From sequence to sequence space. BMC Bioinformatics 9: 394. 18811946 10.1186/1471-2105-9-394 PMC2571980

[22] Dai Q, Yang Y, Wang T (2008) Markov model plus k-word distributions: a synergy that produces novel statistical measures for sequence comparison. Bioinformatics 24: 2296–2302. 18710871 10.1093/bioinformatics/btn436

[23] Qi J, Wang B, Hao B (2004) Whole proteome prokaryote phylogeny without sequence alignment: a K-string composition approach. J Mol Evol 58: 1–11. 14743310 10.1007/s00239-003-2493-7

[24] Song K, Ren J, Zhai Z, Liu X, Deng M, et al (2013) Alignment-Free Sequence Comparison Based on Next-Generation Sequencing Reads. J Comput Biol 20: 64–79. 23383994 10.1089/cmb.2012.0228 PMC3581251

[25] Jiang B, Song K, Ren J, Deng M, Sun F, et al (2012) Comparison of metagenomic samples using sequence signatures. BMC Genomics 13: 730. 23268604 10.1186/1471-2164-13-730 PMC3549735

[26] Pride D, Meinersmann R, Wassenaar T, Blaser M (2003) Evolutionary implications of microbial genome tetranucleotide frequency biases. Genome Res 13: 145–158. 12566393 10.1101/gr.335003 PMC420360

[27] Dalevi D, Dubhashi D, Hermansson M (2006) Bayesian classifiers for detecting HGT using fixed and variable order Markov models of genomic signatures. Bioinformatics 517–522. 10.1093/bioinformatics/btk029 16403797

[28] Teeling H, Meyerdierks A, Bauer M, Amann R, Glöckner F (2004) Application of tetranucleotide frequencies for the assignment of genomic fragments. Environ Microbiol: 938–947. 10.1111/j.1462-2920.2004.00624.x 15305919

[29] Willner D, Thurber R, Rohwer F (2009) Metagenomic signatures of 86 microbial and viral metagenomes. Environ Microbiol 11: 1752–1766. 19302541 10.1111/j.1462-2920.2009.01901.x

[30] Reinert G, Chew D, Sun F (2009) Waterman MS: Alignment-free sequence comparison (I): statistics and power. J Comput Biol 12: 1615–1634. 10.1089/cmb.2009.0198 PMC2818754 20001252

[31] Robinson DR, Foulds LR (1981) Comparison of phylogenetic trees. Math Biosci 53: 131–147.

[32] Robinson DF, LR F (1981) Comparison of phylogenetic trees. Math Biosci 53: 131–147.

[33] Schloss P, Handelsman J (2006) Introducing TreeClimber, a test to compare microbial community structures. Appl Environ Microbiol 72: 2379–2384. 16597933 10.1128/AEM.72.4.2379-2384.2006 PMC1449046

[34] Murtagh F (1984) Complexities of hierarchic clutering algorithms: the state of the art. Comput Stat 1: 101–113.

[35] Anderson M (2003) PCO: a FORTRAN computer program for principal coordinate analysis. New Zealand: Department of Statistics, University of Auckland. 7 p.

[36] Xiong X, Frank D, Robertson C, Hung S, Markle J, et al (2012) Generation and Analysis of a Mouse Intestinal Metatranscriptome through Illumina Based RNA-Sequencing. PLoS ONE 7: e36009. 22558305 10.1371/journal.pone.0036009 PMC3338770

[37] Balzer S, Malde K, Lanzén A, Sharma A, Jonassen I (2010) Characteristics of 454 pyrosequencing data–enabling realistic simulation with flowsim. Bioinformatics 26: 420–425. 10.1093/bioinformatics/btq365 PMC2935434 20823302

[38] Margulies M, Egholm M, Altman WE (2005) etc (2005) Genome sequencing in microfabricated high-density picolitre reactors. Nature 437: 376–380. 16056220 10.1038/nature03959 PMC1464427

[39] Zeng F, Jiang R, Chen T (2013) PyroHMMsnp: a SNP caller for Ion Torrent and 454 sequencing data. Nucl Acid Res 41: e136. 10.1093/nar/gkt372 PMC3711422 23700313

