# Supplementary material for: Comparison of Metatranscriptomic Samples Based on k-Tuple Frequencies
Source: PLoS One. 2014 Jan 2;9(1):e84348. doi: 10.1371/journal.pone.0084348 (PMC3879298; doi:10.1371/journal.pone.0084348)
Supplement: Supplement S1 — Figure S1. The clustering results of both metagenomic and metatranscriptomic datasets in Experiment 3 based on the dissimilarity measures Ch, Hao and Eu. Table S1. Sampling reads number for Experiment 1. Table S2. The symmetric difference between the reference and clustering trees for Experiment 1 (10% sampling). Table S3. The symmetric difference between the reference and clustering trees for Experiment 1 (1% sampling). Table S4. The symmetric difference between the reference and clustering trees for Experiment 1 (0.1% sampling. Table S5. Sampling reads number for Experiment 2. Table S6. The GOF (times 100) by first principle coordinate of PCoA of Experiment 2 (10% sampling). Table S7. The GOF (times 100) by first principle coordinate of PCoA of Experiment 2 (1% samping). Table S8. The GOF (times 100) by first principle coordinate of PCoA of Experiment 2 (0.1% sampling). Table S9. The SRCC between the first principle coordinate and depth for Experiment 2 (10% sampling). Table S10. Sampling reads number for Experiment 3. Table S11. The number of sampling reads for Experiment 4. Table S12. The symmetric difference between the reference and clustering trees in Experiment 4 (1% sampling). Table S13. The symmetric difference between the reference and clustering trees in Experiment 4 (0.1% sampling). Table S14. The symmetric difference between the reference and clustering trees in Experiment 4 (0.01% sampling). (DOC) [file pone.0084348.s001.doc]

**Table S1. The number of sampling reads for Experiment 1**

| Datasets | samples | 10% sampling | 1% sampling | 0.1% sampling |
| --- | --- | --- | --- | --- |
| Hawaii_Aug_DSW_control | Hawaii_Aug_DSW_Control_cDNA_T1 | 50368 | 5037 | 504 |
|  | Hawaii_Aug_DSW_Control_cDNA_T2 | 47739 | 4774 | 478 |
|  | Hawaii_Aug_DSW_Control_cDNA_T3 | 53429 | 5343 | 535 |
|  | Hawaii_Aug_DSW_Control_cDNA_T4 | 59712 | 5972 | 598 |
|  | Hawaii_Aug_DSW_Control_cDNA_T5 | 42981 | 4299 | 430 |
|  | Total | 254229 | 25425 | 2545 |
| Georgia_May | Georgia_May_PACT1 | 11056 | 1106 | 111 |
|  | Georgia_May_PACT2 | 13303 | 1331 | 134 |
|  | Georgia_May_PAPUT1 | 9995 | 1000 | 100 |
|  | Georgia_May_PAPUT2 | 12144 | 1215 | 122 |
|  | Georgia_May_PASPD1 | 11839 | 1184 | 119 |
|  | Georgia_May_PASPD2 | 10447 | 1045 | 105 |
|  | Total | 68784 | 6881 | 691 |
| California_May_Deepsea | California_May_Deepsea_hydrothermalVent_463 | 95457 | 9546 | 955 |
|  | California_May_Deepsea_hydrothermalVent_468 | 75844 | 7585 | 759 |
|  | California_May_Deepsea_salineWater_464 | 57058 | 5706 | 571 |
|  | California_May_Deepsea_salineWater_467 | 54671 | 5468 | 547 |
|  | Total | 283030 | 28305 | 2832 |
| WesternEnglish_season_Aug | WesternEnglish_season_Aug_10am | 8584 | 859 | 86 |
|  | WesternEnglish_season_Aug_10pm | 19328 | 1933 | 194 |
|  | WesternEnglish_season_Aug_4am | 15490 | 1549 | 155 |
|  | WesternEnglish_season_Aug_4pm | 13940 | 1394 | 140 |
|  | Total | 57342 | 5735 | 575 |

**Table S2The symmetric difference between the reference and clustering trees in Experiment 1** (10% sampling)

| *k* | 2 | 3 | 4 | 5 | 6 | 7 | 8 | 9 | 10 |
| --- | --- | --- | --- | --- | --- | --- | --- | --- | --- |
| $d_{2}$ | 15.1 | 15.5 | 16.0 | 15.5 | 14.7 | 14.0 | 13.5 | 13.2 | 13.0 |
| $d_{2}^{s}$|M0 | 17.6 | 14.7 | 15.3 | 14.8 | 13.0 | 12.3 | 12.1 | 12.0 | 12.0 |
| $d_{2}^{s}$|M1 | 14.9 | 15.3 | 15.2 | 13.9 | 12.8 | 12.2 | 12.1 | 12.0 | 12.3 |
| $d_{2}^{s}$|M2 | NA | 14.1 | 15.6 | 14.8 | 12.7 | 12.3 | 12.1 | 12.0 | 12.1 |
| $d_{2}^{s}$|M3 | NA | NA | 14.8 | 13.6 | 12.6 | 12.3 | 12.0 | 12.0 | 12.2 |
| $d_{2}^{*}$|M0 | 17.8 | 15.1 | 15.7 | 15.4 | 13.5 | 13.0 | 12.8 | 12.9 | 13.0 |
| $d_{2}^{*}$|M1 | 15.0 | 14.8 | 15.6 | 14.6 | 13.0 | 13.0 | 12.7 | 12.7 | 12.2 |
| $d_{2}^{*}$|M2 | NA | 14.5 | 15.7 | 15.0 | 13.5 | 13.0 | 12.7 | 12.4 | 12.0 |
| $d_{2}^{*}$|M3 | NA | NA | 13.9 | 13.6 | 12.5 | 12.3 | 12.0 | 12.1 | 12.1 |
| $S2$|M0 | 22.0 | 22.0 | 18.0 | 18.0 | 18.0 | 18.0 | 16.5 | 16.0 | 18.6 |
| $S2$|M1 | 22.0 | 20.3 | 18.0 | 18.0 | 18.0 | 18.0 | 17.4 | 16.0 | 15.7 |
| $S2$|M2 | NA | 18.0 | 18.0 | 18.0 | 18.0 | 18.0 | 17.9 | 16.0 | 15.0 |
| *Hao* | NA | 14.8 | 15.7 | 13.4 | 12.2 | 12.4 | 12.1 | 14.7 | 15.0 |
| *Ma* | 15.3 | 15.3 | 15.9 | 17.8 | 15.9 | 12.6 | 12.2 | 12.1 | 12.0 |
| *Eu* | 15.5 | 15.6 | 17.8 | 17.6 | 16.7 | 16.2 | 15.7 | 15.3 | 15.3 |
| *Ch* | 15.5 | 21.4 | 19.6 | 21.0 | 18.8 | 20.9 | 18.9 | 20.9 | 20.9 |

*For score <20, p-value<0.001; 20≦score<22, p-value=0.004; score=22, p-value=0.098

**Table S3The symmetric difference between the reference and clustering trees in Experiment 1** (1% sampling)

| *k* | 2 | 3 | 4 | 5 | 6 | 7 | 8 | 9 | 10 |
| --- | --- | --- | --- | --- | --- | --- | --- | --- | --- |
| $d_{2}$ | 15.5 | 15.2 | 16.0 | 14.6 | 14.6 | 14.2 | 14.4 | 14.4 | 14.5 |
| $d_{2}^{s}$|M0 | 18.1 | 14.7 | 14.9 | 14.2 | 13.6 | 13.6 | 14.0 | 14.0 | 14.1 |
| $d_{2}^{s}$|M1 | 15.2 | 15.2 | 15.0 | 14.1 | 13.7 | 13.9 | 13.9 | 14.0 | 14.0 |
| $d_{2}^{s}$|M2 | NA | 14.9 | 15.2 | 14.1 | 13.8 | 14.0 | 13.9 | 14.0 | 14.1 |
| $d_{2}^{s}$|M3 | NA | NA | 15.4 | 13.5 | 13.4 | 13.7 | 13.8 | 14.0 | 14.0 |
| $d_{2}^{*}$|M0 | 17.5 | 15.0 | 15.5 | 14.8 | 14.4 | 14.3 | 14.3 | 14.3 | 14.5 |
| $d_{2}^{*}$|M1 | 15.2 | 15.2 | 15.0 | 14.6 | 14.1 | 13.8 | 13.6 | 13.4 | 13.4 |
| $d_{2}^{*}$|M2 | NA | 15.6 | 15.1 | 14.2 | 14.0 | 13.7 | 13.5 | 13.4 | 13.4 |
| $d_{2}^{*}$|M3 | NA | NA | 15.4 | 13.6 | 13.4 | 13.2 | 13.2 | 13.2 | 13.5 |
| $S2$|M0 | 21.8 | 21.7 | 18.0 | 18.0 | 18.0 | 17.7 | 16.9 | 16.0 | 16.0 |
| $S2$|M1 | 21.8 | 20.6 | 18.0 | 18.0 | 18.0 | 17.5 | 16.2 | 16.0 | 16.0 |
| $S2$|M2 | NA | 18.0 | 18.0 | 18.0 | 18.0 | 17.5 | 17.5 | 16.0 | 16.0 |
| *Hao* | NA | 15.4 | 15.0 | 13.8 | 13.9 | 14.0 | 14.0 | 14.2 | 17.9 |
| *Ma* | 15.3 | 15.8 | 15.8 | 16.3 | 15.4 | 14.3 | 13.3 | 13.6 | 13.8 |
| *Eu* | 15.6 | 15.8 | 16.7 | 16.7 | 16.4 | 16.1 | 16.0 | 16.0 | 16.0 |
| *Ch* | 15.3 | 18.7 | 19.7 | 20.2 | 19.6 | 21.5 | 20.3 | 21.6 | 21.6 |

*For score <20, p-value<0.001; 20≦score<22, p-value=0.004; score=22, p-value=0.098

**Table S4The symmetric difference between the reference and clustering trees in Experiment 1** (0.1% sampling)

| *k* | 2 | 3 | 4 | 5 | 6 | 7 | 8 | 9 | 10 |
| --- | --- | --- | --- | --- | --- | --- | --- | --- | --- |
| $d_{2}$ | 18.1 | 16.8 | 16.1 | 15.6 | 14.9 | 14.9 | 14.8 | 14.9 | 15.0 |
| $d_{2}^{s}$|M0 | 18.9 | 15.4 | 14.9 | 14.8 | 14.6 | 14.8 | 14.6 | 14.6 | 14.8 |
| $d_{2}^{s}$|M1 | 18.9 | 15.4 | 15.3 | 14.7 | 14.7 | 14.8 | 14.5 | 14.6 | 14.9 |
| $d_{2}^{s}$|M2 | NA | 17.0 | 15.0 | 14.7 | 14.6 | 14.8 | 14.6 | 14.6 | 14.8 |
| $d_{2}^{s}$|M3 | NA | NA | 16.3 | 14.7 | 14.6 | 14.8 | 14.6 | 14.6 | 14.7 |
| $d_{2}^{*}$|M0 | 18.8 | 16.0 | 15.2 | 15.0 | 14.8 | 14.8 | 14.7 | 14.8 | 14.7 |
| $d_{2}^{*}$|M1 | 18.5 | 16.1 | 15.3 | 14.7 | 14.7 | 14.7 | 14.6 | 14.6 | 14.6 |
| $d_{2}^{*}$|M2 | NA | 17.8 | 15.3 | 14.7 | 14.6 | 14.6 | 14.7 | 14.6 | 14.6 |
| $d_{2}^{*}$|M3 | NA | NA | 16.4 | 14.8 | 14.6 | 14.6 | 14.5 | 14.5 | 14.5 |
| $S2$|M0 | 22.0 | 21.2 | 18.1 | 18.0 | 18.0 | 17.2 | 16.4 | 17.3 | 21.1 |
| $S2$|M1 | 21.8 | 20.3 | 17.9 | 17.8 | 18.0 | 17.4 | 16.4 | 17.3 | 21.1 |
| $S2$|M2 | NA | 18.8 | 18.0 | 17.9 | 18.0 | 17.7 | 17.4 | 17.4 | 21.2 |
| *Hao* | NA | 15.9 | 15.6 | 15.0 | 14.8 | 15.0 | 17.1 | 19.8 | 23.8 |
| *Ma* | 18.0 | 16.5 | 15.9 | 16.6 | 16.8 | 16.4 | 14.9 | 14.8 | 14.8 |
| *Eu* | 18.2 | 17.0 | 16.7 | 16.9 | 16.9 | 17.0 | 17.0 | 17.0 | 17.0 |
| *Ch* | 18.0 | 18.8 | 19.1 | 20.1 | 20.3 | 20.61 | 20.41 | 20.61 | 20.41 |

*For score <20, p-value<0.001; 20≦score<22, p-value=0.004; score=22, p-value=0.098

**Table S5 The number of sampling reads for Experiment 2**

| Dataset | samples | 10% sampling | 1% sampling | 0.1% sampling |
| --- | --- | --- | --- | --- |
| NPSG  (MT) | NPSG_MT_25m_1 | 4447 | 445 | 45 |
|  | NPSG_MT_25m_2 | 4283 | 429 | 43 |
|  | NPSG_MT_75m_1 | 6361 | 637 | 64 |
|  | NPSG_MT_75m_2 | 6327 | 633 | 64 |
|  | NPSG_MT_125m_1 | 5808 | 581 | 59 |
|  | NPSG_MT_125m_2 | 5699 | 570 | 57 |
|  | NPSG_MT_500m_1 | 6044 | 605 | 61 |
|  | NPSG_MT_500m_2 | 6182 | 619 | 62 |
|  | Total | 45151 | 4519 | 455 |

**Table S6TheGOF (times 100) by the first principal coordinate of PCoAin Experiment 2 (10%)**

| *k* | 2 | 3 | 4 | 5 | 6 | 7 | 8 | 9 | 10 |
| --- | --- | --- | --- | --- | --- | --- | --- | --- | --- |
| $d_{2}$ | 81 | 79 | 80 | 80 | 80 | 81 | 82 | 83 | 83 |
| $d_{2}^{s}$|M0 | 85 | 84 | 77 | 74 | 74 | 74 | 71 | 63 | 49 |
| $d_{2}^{s}$|M1 | 69 | 71 | 76 | 75 | 75 | 73 | 69 | 61 | 48 |
| $d_{2}^{s}$|M2 | NA | 39 | 80 | 77 | 75 | 73 | 69 | 60 | 48 |
| $d_{2}^{s}$|M3 | NA | NA | 60 | 74 | 77 | 74 | 69 | 61 | 49 |
| $d_{2}^{*}$|M0 | 82 | 82 | 79 | 73 | 69 | 67 | 62 | 58 | 56 |
| $d_{2}^{*}$|M1 | 76 | 69 | 76 | 78 | 80 | 81 | 82 | 82 | 82 |
| $d_{2}^{*}$|M2 | NA | 69 | 82 | 82 | 83 | 83 | 83 | 83 | 83 |
| $d_{2}^{*}$|M3 | NA | NA | 52 | 81 | 82 | 84 | 86 | 87 | 89 |
| $S2$|M0 | 69 | 59 | 42 | 18 | 15 | 14 | 14 | 14 | 14 |
| $S2$|M1 | 73 | 78 | 78 | 32 | 16 | 15 | 14 | 14 | 14 |
| $S2$|M2 | NA | 82 | 86 | 55 | 22 | 16 | 14 | 14 | 14 |
| *Hao* | NA | 62 | 80 | 79 | 84 | 80 | 57 | 28 | 21 |
| *Ma* | 78 | 65 | 59 | 60 | 63 | 62 | 57 | 51 | 45 |
| *Eu* | 84 | 84 | 90 | 93 | 95 | 95 | 95 | 95 | 95 |
| *Ch* | 98 | 98 | 99 | 99 | 99 | 99 | 99 | 99 | 99 |

*For correlation coefficient $>=0.7$, the p-value<0.03

**Table S7TheGOF (times 100) by the first principal coordinate of PCoAin Experiment 2 (1%)**

| *k* | 2 | 3 | 4 | 5 | 6 | 7 | 8 | 9 | 10 |
| --- | --- | --- | --- | --- | --- | --- | --- | --- | --- |
| $d_{2}$ | 81 | 80 | 80 | 81 | 81 | 80 | 79 | 78 | 75 |
| $d_{2}^{s}$|M0 | 86 | 79 | 66 | 57 | 53 | 46 | 37 | 31 | 30 |
| $d_{2}^{s}$|M1 | 65 | 67 | 67 | 58 | 53 | 45 | 36 | 31 | 29 |
| $d_{2}^{s}$|M2 | NA | 31 | 66 | 58 | 52 | 45 | 36 | 31 | 30 |
| $d_{2}^{s}$|M3 | NA | NA | 39 | 53 | 52 | 45 | 36 | 31 | 30 |
| $d_{2}^{*}$|M0 | 82 | 78 | 73 | 63 | 57 | 54 | 51 | 50 | 50 |
| $d_{2}^{*}$|M1 | 71 | 63 | 70 | 70 | 68 | 68 | 69 | 68 | 67 |
| $d_{2}^{*}$|M2 | NA | 62 | 77 | 75 | 72 | 70 | 69 | 67 | 66 |
| $d_{2}^{*}$|M3 | NA | NA | 40 | 67 | 64 | 62 | 61 | 60 | 59 |
| $S2$|M0 | 70 | 48 | 18 | 15 | 14 | 14 | 14 | 14 | 14 |
| $S2$|M1 | 74 | 72 | 30 | 16 | 15 | 14 | 14 | 14 | 14 |
| $S2$|M2 | NA | 78 | 43 | 20 | 15 | 14 | 14 | 14 | 14 |
| *Hao* | NA | 59 | 74 | 63 | 53 | 34 | 20 | 20 | 23 |
| *Ma* | 76 | 60 | 53 | 49 | 45 | 39 | 33 | 31 | 29 |
| *Eu* | 82 | 81 | 87 | 90 | 91 | 91 | 91 | 90 | 89 |
| *Ch* | 95 | 97 | 98 | 99 | 99 | 99 | 99 | 99 | 99 |

**Table S8TheGOF (times 100) by the first principal coordinate of PCoAin Experiment 2 (0.1%)**

| *k* | 2 | 3 | 4 | 5 | 6 | 7 | 8 | 9 | 10 |
| --- | --- | --- | --- | --- | --- | --- | --- | --- | --- |
| $d_{2}$ | 79 | 76 | 71 | 58 | 39 | 31 | 31 | 31 | 30 |
| $d_{2}^{s}$|M0 | 59 | 33 | 27 | 24 | 23 | 22 | 21 | 21 | 21 |
| $d_{2}^{s}$|M1 | 31 | 30 | 27 | 24 | 23 | 21 | 21 | 20 | 20 |
| $d_{2}^{s}$|M2 | NA | 23 | 26 | 24 | 22 | 21 | 21 | 20 | 20 |
| $d_{2}^{s}$|M3 | NA | NA | 20 | 23 | 22 | 21 | 20 | 20 | 20 |
| $d_{2}^{*}$|M0 | 46 | 29 | 29 | 30 | 30 | 31 | 31 | 31 | 30 |
| $d_{2}^{*}$|M1 | 37 | 35 | 31 | 29 | 27 | 26 | 26 | 26 | 25 |
| $d_{2}^{*}$|M2 | NA | 27 | 31 | 28 | 27 | 26 | 25 | 25 | 24 |
| $d_{2}^{*}$|M3 | NA | NA | 27 | 26 | 24 | 23 | 23 | 23 | 22 |
| $S2$|M0 | 60 | 20 | 15 | 14 | 14 | 14 | 14 | 14 | 14 |
| $S2$|M1 | 66 | 27 | 16 | 14 | 14 | 14 | 14 | 14 | 14 |
| $S2$|M2 | NA | 32 | 18 | 15 | 14 | 14 | 14 | 14 | 14 |
| *Hao* | NA | 38 | 30 | 25 | 19 | 17 | 20 | 22 | 22 |
| *Ma* | 56 | 38 | 29 | 24 | 21 | 20 | 20 | 20 | 20 |
| *Eu* | 60 | 57 | 60 | 61 | 62 | 61 | 59 | 57 | 55 |
| *Ch* | 70 | 81 | 85 | 87 | 88 | 89 | 90 | 90 | 91 |

**Table S9TheSRCCbetween the first principal coordinate and depth in Experiment 2(10% sampling)**

| *K* | 2 | 3 | 4 | 5 | 6 | 7 | 8 | 9 | 10 |
| --- | --- | --- | --- | --- | --- | --- | --- | --- | --- |
| $d_{2}$ | 0.7807 | 0.7807 | 0.3904 | 0.3904 | 0.3904 | 0.5855 | 0.7807 | 0.7807 | 0.7807 |
| $d_{2}^{s}$|M0 | 0.3904 | 0.3904 | 0.7807 | 0.7807 | 0.7807 | 0.9759 | 0.7807 | 0.7807 | 0.7807 |
| $d_{2}^{s}$|M1 | 0.3904 | 0.3904 | 0.7807 | 0.7807 | 0.7807 | 0.7807 | 0.7807 | 0.8259 | 0.7807 |
| $d_{2}^{s}$|M2 | NA | 0 | 0.7807 | 0.7807 | 0.7807 | 0.7807 | 0.7807 | 0.7807 | 0.7807 |
| $d_{2}^{s}$|M3 | NA | NA | 0.6831 | 0.7807 | 0.7807 | 0.7807 | 0.7807 | 0.7807 | 0.7807 |
| $d_{2}^{*}$|M0 | 0.4880 | 0.3904 | 0.3904 | 0.3904 | 0.3904 | 0.3904 | 0.1952 | 0.3904 | 0.3904 |
| $d_{2}^{*}$|M1 | 0.5855 | 0.7807 | 0.7807 | 0.7807 | 0.7807 | 0.7807 | 0.7807 | 0.7807 | 0.7807 |
| $d_{2}^{*}$|M2 | NA | 0.2928 | 0.7807 | 0.7807 | 0.7807 | 0.7807 | 0.7807 | 0.7807 | 0.7807 |
| $d_{2}^{*}$|M3 | NA | NA | 0.5855 | 0.7807 | 0.7807 | 0.7807 | 0.7807 | 0.7807 | 0.7807 |
| $S2$|M0 | 0.3904 | 0.1952 | 0.3904 | 0.3904 | 0.3904 | 0.5855 | 0.3904 | 0.0976 | 0.4392 |
| $S2$|M1 | 0.1952 | 0.1952 | 0.3904 | 0.3904 | 0.3904 | 0.3904 | 0.3904 | 0.3904 | 0.0976 |
| $S2$|M2 | NA | 0.1952 | 0.0976 | 0.3904 | 0.3904 | 0.3904 | 0.3904 | 0.3904 | 0.1952 |
| *Hao* | NA | 0.9760 | 0.7807 | 0.7807 | 0.7807 | 0.9759 | 0.9271 | 0.7807 | 0.3904 |
| *Ma* | 0.7807 | 0.7807 | 0.7807 | 0.7807 | 0.7807 | 0.7807 | 0.7807 | 0.7807 | 0.7807 |
| *Eu* | 0.7807 | 0.7807 | 0.3904 | 0.3904 | 0.3904 | 0.3904 | 0.3904 | 0.3904 | 0.3904 |
| *Ch* | 0.7807 | 0.5855 | 0.3904 | 0.3904 | 0.3904 | 0.3904 | 0.3904 | 0.3904 | 0.3904 |

**Table S10The number of sampling reads in Experiment 3**

| Datasets | samples | 10% sampling | 1% sampling | 0.1% sampling |
| --- | --- | --- | --- | --- |
| NPSG_depth | NPSG_MT_25m_1 | 4447 | 445 | 45 |
|  | NPSG_MT_25m_2 | 4283 | 429 | 43 |
|  | NPSG_MT_75m_1 | 6361 | 637 | 64 |
|  | NPSG_MT_75m_2 | 6327 | 633 | 64 |
|  | NPSG_MT_125m_1 | 5808 | 581 | 59 |
|  | NPSG_MT_125m_2 | 5699 | 570 | 57 |
|  | NPSG_MT_500m_1 | 6044 | 605 | 61 |
|  | NPSG_MT_500m_2 | 6182 | 619 | 62 |
|  | NPSG_MG_25m_1 | 18610 | 1861 | 187 |
|  | NPSG_MG_25m_2 | 19512 | 1952 | 196 |
|  | NPSG_MG_75m_1 | 20864 | 2096 | 209 |
|  | NPSG_MG_75m_2 | 20056 | 2006 | 201 |
|  | NPSG_MG_125m_1 | 18514 | 1852 | 186 |
|  | NPSG_MG_125m_2 | 16326 | 1633 | 164 |
|  | NPSG_MG_500m_1 | 19135 | 1914 | 192 |
|  | NPSG_MG_500m_2 | 19627 | 1963 | 197 |
|  | Total | 197795 | 19787 | 1987 |


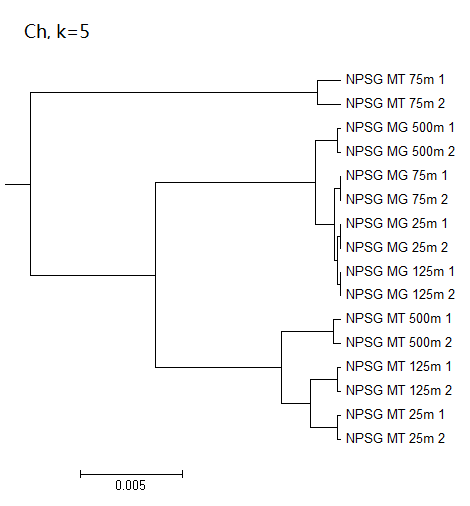

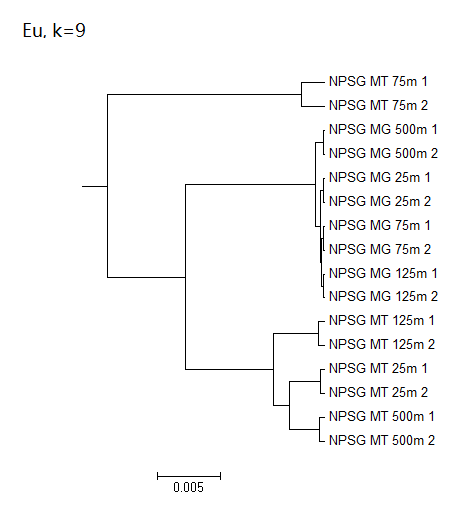


1. (b)


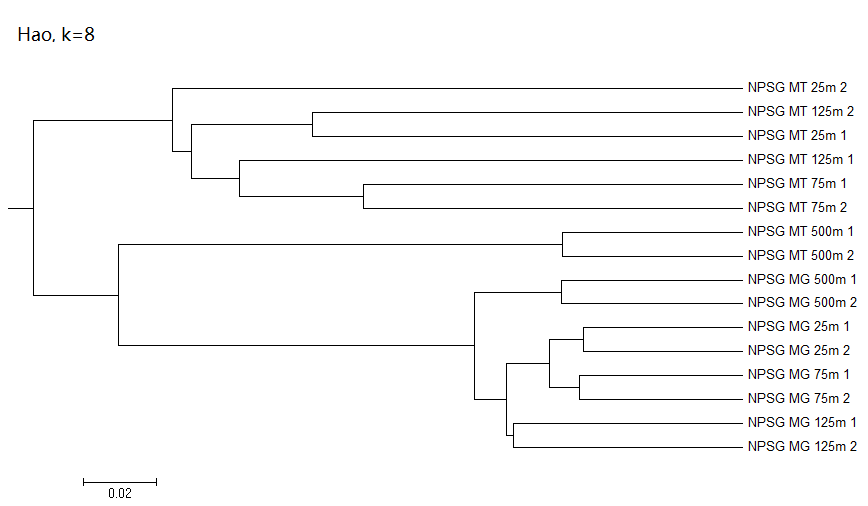


(c)

**Figure S1 The clustering results of both metagenomic and metatranscriptomic datasets in Experiment 3 based on the dissimilarity measures Ch, Hao and Eu.**

**Table S11The number of sampling reads for Experiment 4**

| Datasets | samples | 1% sampling | 0.1% sampling | 0.01% sampling |
| --- | --- | --- | --- | --- |
| Mouse_Intestinal | NOD501CecQN | 14631 | 1464 | 147 |
|  | NOD501ColQN | 13343 | 1335 | 134 |
|  | NOD502CecQN | 21784 | 2179 | 218 |
|  | NOD502ColQN | 11491 | 1150 | 115 |
|  | NOD503CecQN | 16263 | 1627 | 163 |
|  | NOD504CecQN | 19861 | 1987 | 199 |
|  | NOD504ColQN | 17101 | 1711 | 172 |
|  | Total | 114474 | 11453 | 1148 |

**Table S12The symmetric difference between the reference and clustering trees in Experiment 4 (1% sampling)**

*score=5, p-value=0.043; score=7, p-value=1.0

| *k* | 2 | 3 | 4 | 5 | 6 | 7 | 8 | 9 | 10 |
| --- | --- | --- | --- | --- | --- | --- | --- | --- | --- |
| $d_{2}$ | 7 | 7 | 7 | 7 | 7 | 7 | 7 | 7 | 7 |
| $d_{2}^{s}$|M0 | 7 | 7 | 5 | 5 | 5 | 5 | 5 | 5 | 5 |
| $d_{2}^{s}$|M1 | 7 | 7 | 7 | 6 | 5 | 5 | 5 | 5 | 5 |
| $d_{2}^{s}$|M2 | NA | 7 | 7 | 7 | 6 | 5 | 5 | 5 | 5 |
| $d_{2}^{s}$|M3 | NA | NA | 7 | 7 | 7 | 5 | 5 | 5 | 5 |
| $d_{2}^{*}$|M0 | 7 | 7 | 7 | 7 | 7 | 7 | 7 | 7 | 7 |
| $d_{2}^{*}$|M1 | 7 | 7 | 7 | 7 | 7 | 7 | 7 | 7 | 7 |
| $d_{2}^{*}$|M2 | NA | 7 | 7 | 7 | 7 | 7 | 7 | 7 | 7 |
| $d_{2}^{*}$|M3 | NA | NA | 7 | 7 | 7 | 7 | 7 | 7 | 7 |
| $S2$|M0 | 7 | 7 | 7 | 7 | 6 | 5 | 7 | 7 | 7 |
| $S2$|M1 | 7 | 7 | 7 | 7 | 5 | 5 | 7 | 7 | 7 |
| $S2$|M2 | NA | 7 | 7 | 7 | 5 | 5 | 5 | 7 | 7 |
| *Hao* | NA | 7 | 7 | 7 | 5 | 5 | 7 | 7 | 7 |
| *Ma* | 7 | 7 | 7 | 6 | 5 | 5 | 5 | 5 | 5 |
| *Eu* | 7 | 7 | 7 | 7 | 7 | 7 | 7 | 7 | 7 |
| *Ch* | 7 | 6 | 7 | 7 | 7 | 7 | 7 | 7 | 7 |

**Table S13The symmetric difference between the reference and clustering trees in Experiment 4 (0.**1% sampling)

| *k* | 2 | 3 | 4 | 5 | 6 | 7 | 8 | 9 | 10 |
| --- | --- | --- | --- | --- | --- | --- | --- | --- | --- |
| $d_{2}$ | 7 | 7 | 7 | 7 | 7 | 7 | 7 | 7 | 7 |
| $d_{2}^{s}$|M0 | 7 | 6 | 5 | 6 | 5 | 5 | 5 | 5 | 5 |
| $d_{2}^{s}$|M1 | 7 | 7 | 7 | 6 | 5 | 5 | 5 | 5 | 5 |
| $d_{2}^{s}$|M2 | NA | 7 | 7 | 7 | 6 | 5 | 5 | 5 | 5 |
| $d_{2}^{s}$|M3 | NA | NA | 7 | 7 | 6 | 5 | 5 | 5 | 5 |
| $d_{2}^{*}$|M0 | 7 | 7 | 6 | 6 | 7 | 7 | 7 | 7 | 7 |
| $d_{2}^{*}$|M1 | 7 | 7 | 7 | 7 | 7 | 7 | 7 | 7 | 7 |
| $d_{2}^{*}$|M2 | NA | 7 | 7 | 7 | 7 | 7 | 7 | 7 | 7 |
| $d_{2}^{*}$|M3 | NA | NA | 7 | 7 | 7 | 7 | 7 | 7 | 7 |
| $S2$|M0 | 7 | 7 | 7 | 7 | 5 | 6 | 7 | 7 | 7 |
| $S2$|M1 | 7 | 7 | 7 | 7 | 6 | 5 | 7 | 7 | 7 |
| $S2$|M2 | NA | 7 | 7 | 7 | 5 | 5 | 7 | 7 | 7 |
| *Hao* | NA | 7 | 7 | 7 | 5 | 6 | 7 | 6 | 7 |
| *Ma* | 7 | 7 | 7 | 7 | 6 | 5 | 5 | 5 | 5 |
| *Eu* | 7 | 7 | 7 | 7 | 7 | 7 | 7 | 7 | 7 |
| *Ch* | 7 | 7 | 7 | 7 | 7 | 7 | 7 | 7 | 7 |

*score=5, p-value=0.043; score=7, p-value=1.0

**Table S14The symmetric difference between the reference and clustering trees in Experiment 4 (0.0**1% sampling)

| *k* | 2 | 3 | 4 | 5 | 6 | 7 | 8 | 9 | 10 |
| --- | --- | --- | --- | --- | --- | --- | --- | --- | --- |
| $d_{2}$ | 6 | 7 | 7 | 7 | 6 | 6 | 6 | 6 | 6 |
| $d_{2}^{s}$|M0 | 7 | 6 | 6 | 6 | 5 | 5 | 5 | 5 | 5 |
| $d_{2}^{s}$|M1 | 7 | 7 | 6 | 6 | 5 | 5 | 5 | 5 | 5 |
| $d_{2}^{s}$|M2 | NA | 7 | 6 | 6 | 5 | 5 | 5 | 5 | 5 |
| $d_{2}^{s}$|M3 | NA | NA | 7 | 6 | 6 | 5 | 5 | 5 | 5 |
| $d_{2}^{*}$|M0 | 7 | 6 | 6 | 6 | 6 | 6 | 6 | 6 | 6 |
| $d_{2}^{*}$|M1 | 7 | 7 | 7 | 6 | 6 | 6 | 6 | 6 | 6 |
| $d_{2}^{*}$|M2 | NA | 7 | 6 | 6 | 6 | 6 | 7 | 7 | 7 |
| $d_{2}^{*}$|M3 | NA | NA | 6 | 6 | 6 | 6 | 6 | 6 | 6 |
| $S2$|M0 | 7 | 7 | 7 | 7 | 7 | 7 | 7 | 7 | 7 |
| $S2$|M1 | 7 | 7 | 7 | 7 | 7 | 7 | 7 | 7 | 7 |
| $S2$|M2 | NA | 7 | 7 | 7 | 7 | 7 | 7 | 7 | 7 |
| *Hao* | NA | 7 | 6 | 6 | 6 | 6 | 7 | 7 | 7 |
| *Ma* | 7 | 7 | 7 | 7 | 6 | 6 | 5 | 5 | 5 |
| *Eu* | 7 | 7 | 7 | 7 | 7 | 7 | 7 | 7 | 7 |
| *Ch* | 7 | 7 | 7 | 7 | 6 | 7 | 7 | 7 | 7 |

*score=5, p-value=0.043; score=7, p-value=1.0
